# Enhanced accumulation of oil through co-expression of fatty acid and ABC transporters in *Chlamydomonas* under standard growth conditions

**DOI:** 10.1186/s13068-022-02154-6

**Published:** 2022-05-20

**Authors:** Ru Chen, Miao Yang, Mengjie Li, Hao Zhang, Han Lu, Xiaotan Dou, Shiqi Feng, Song Xue, Chenba Zhu, Zhanyou Chi, Fantao Kong

**Affiliations:** 1 School of Bioengineering, Dalian University of Technology, Dalian, 116024 China; 2 School of Life Science, Ganjingzi District, Liaoning Normal University, No. 1, Liushu South Street, Dalian, 116081 China; 3 Institute of Biotechnology, RWTH Aachen University, Worringerweg 3, 52074 Aachen, Germany

**Keywords:** *Chlamydomonas reinhardtii*, Genetic engineering, Fatty acid exporters, ABC transporter, Triacylglycerol, Polyunsaturated fatty acids

## Abstract

**Background:**

Chloroplast and endoplasmic reticulum (ER)-localized fatty acid (FA) transporters have been reported to play important roles in oil (mainly triacylglycerols, TAG) biosynthesis. However, whether these FA transporters synergistically contribute to lipid accumulation, and their effect on lipid metabolism in microalgae are unknown.

**Results:**

Here, we co-overexpressed two chloroplast-localized FA exporters (*FAX1* and *FAX2*) and one ER-localized FA transporter (*ABCA2*) in *Chlamydomonas*. Under standard growth conditions, *FAX1*/*FAX2*/*ABCA2* over-expression lines (OE) accumulated up to twofold more TAG than the parental strain UVM4, and the total amounts of major polyunsaturated FAs (PUFA) in TAG increased by 4.7-fold. In parallel, the total FA contents and major membrane lipids in *FAX1*/*FAX2*/*ABCA2-*OE also significantly increased compared with those in the control lines. Additionally, the total accumulation contribution ratio of PUFA, to total FA and TAG synthesis in *FAX1*/*FAX2*/*ABCA2-*OE, was 54% and 40% higher than that in UVM4, respectively. Consistently, the expression levels of genes directly involved in TAG synthesis, such as type-II diacylglycerol acyltransferases (*DGTT1*, *DGTT3* and *DGTT5*), and phospholipid:diacylglycerol acyltransferase 1 (*PDAT1*), significantly increased, and the expression of *PGD1* (MGDG-specific lipase) was upregulated in *FAX1*/*FAX2*/*ABCA2-*OE compared to UVM4.

**Conclusion:**

These results indicate that the increased expression of *FAX1*/*FAX2*/*ABCA2* has an additive effect on enhancing TAG, total FA and membrane lipid accumulation and accelerates the PUFA remobilization from membrane lipids to TAG by fine-tuning the key genes involved in lipid metabolism under standard growth conditions. Overall, *FAX1*/*FAX2/ABCA2*-OE shows better traits for lipid accumulation than the parental line and previously reported individual FA transporter-OE. Our study provides a potential useful strategy to increase the production of FA-derived energy-rich and value-added compounds in microalgae.

**Supplementary Information:**

The online version contains supplementary material available at 10.1186/s13068-022-02154-6.

## Background

Microalgae are a group of photosynthetic microorganisms, that can convert CO_2_ and light energy into high value-added products and energy-rich reserve compounds such as oils (mainly triacylglycerols, TAG) [1]. They are considered as one of the most promising platforms to produce biofuels. However, the oil content in most microalgal species is naturally low, since most microalgae accumulate large amounts of oils only when subjected to stress conditions (e.g., nutrient depletion and high light) [2, 3]. At present, the industrial production of microalgal biofuels is not economically viable. One solution is to increase the oil productivity by genetic engineering approaches, which require a comprehensive understanding of lipid metabolism [4].

The green microalga *Chlamydomonas reinhardtii* (hereafter referred to as *Chlamydomonas*) is an established algal model due to its fully sequenced genome, high transformation efficiency, and versatile molecular tools available for this organism. *Chlamydomonas* has also emerged as a model organism to study the molecular mechanism of oil accumulation [5, 6]. Over the past decade, lipid metabolism has been modified through main approaches, such as enhancing the TAG biosynthesis, manipulating transcription factors/regulators, blocking competing pathways, modulating the redox state and reducing lipid degradation. Among those approaches, much of the research effort has focused on engineering the key enzymes in the lipid biosynthetic pathway in *Chlamydomonas* [4, 7]. For example, the over-expression of plastidial *LPAAT1* (lysophosphatidic acid acyltransferase 1) or endoplasmic reticulum (ER)-located *LPAAT2* was reported to increase the oil content (> 20%) in *Chlamydomonas* under nitrogen (N)-depleted conditions [8, 9]. However, the effect of the *LPAAT2* over-expression on the TAG synthesis in *Chlamydomonas* under standard growth conditions remains negligible [8]. Diacylglycerol acyltransferases (DGAT) catalyse the last acylation step of the lipid biosynthetic pathway and convert diacylglycerol to TAG [10]. *Chlamydomonas* has six DGAT isozymes, which are classified into two types: type-I DGTA (DGAT1) and type-II DGAT (DGTT1-5) [11]. It was reported that over-expression of *DGTT4* can enhance the TAG accumulation under phosphorus deprivation [12], while the over-expression of three of the five other type-II *DGATs* in *Chlamydomonas* showed no effects on the intracellular TAG levels under standard growth or stress conditions [13]. These results suggest that the over-expression of the key genes can be a strategy to enhance the TAG biosynthesis under certain conditions but does not always work as expected. Moreover, most of the mutants generated through those strategies can accumulate quantities of oils only under stress conditions when the biomass is compromised [4, 14]. Therefore, more alternative approaches are required to promote oil production from microalgae at cost efficiency.

In plants and microalgae, TAG biosynthesis requires substantial fatty acids (FA) as building blocks, which are synthesized in plastids and subsequently transported to the endoplasmic reticulum (ER) for modification [15]. The FA transporters involved in cellular lipid transport were reported to play critical roles in the lipid metabolism in *Chlamydomonas* [16, 17]. At present, two chloroplast inner-membrane-located FA exporters (FAX1 and FAX2) have been identified in *Chlamydomonas* [17]. The over-expression of either *FAX1* or *FAX2* in the *Chlamydomonas* UVM4 strain increased the TAG accumulation (up to 38%) under standard (N-replete) growth conditions [17]. Recently, we identified one of the ATP-binding cassette (ABC) transporters of subfamily A in *Chlamydomonas*, ABCA2, which is located in the ER and involved in the FA transport. The over-expression lines (OE) of *ABCA2* exhibited higher TAG levels under standard growth conditions in *Chlamydomonas* [16]. Therefore, manipulating the expression of FA transporter genes is another promising alternative strategy to accelerate the FA transport from the chloroplast to the ER for TAG biosynthesis. However, individual expression of *FAX1*, *FAX2* or *ABCA2* can only result in a limited increase in oil contents (< 50%) [16, 17], and whether FA transporters synergistically contribute to the lipid accumulation in microalgae was unknown.

To investigate the function and effect of coordinate expression of chloroplast- and ER-located FA transporters on lipid metabolism in microalgae, we co-expressed *FAX1*, *FAX2*, and *ABCA2* in *Chlamydomonas* in this study. *FAX1*/*FAX2*/*ABCA2*-OE showed enhanced accumulation of TAG and increased total FA and major membrane lipids relative to the parental line and individual FA transporter-OE under standard growth conditions. Additionally, the total accumulation contribution ratio of major polyunsaturated FAs (PUFA) to total FA and TAG synthesis in *FAX1*/*FAX2*/*ABCA2-*OE, was higher than that in the parental strain UVM4, and these increased PUFA could be remobilized from membrane lipids to TAG in *FAX1*/*FAX2*/*ABCA2-*OE. Consistently, the expression of key genes in de novo TAG synthesis and membrane lipid-derived TAG synthesis was significantly upregulated in the *FAX1*/*FAX2*/*ABCA2-*OE cells. Overall, our results indicate that the simultaneously increased expression of *FAX1*, *FAX2* and *ABCA2* has an additive effect on enhancing the TAG, total FA and membrane lipid accumulation, and favours the integration of PUFA from membrane lipids to TAG. The *FAX1*/*FAX2/ABCA2*-OE generated in this study showed better traits for lipid accumulation than previously reported individual FA transporter-OE. Therefore, this study provides additional molecular tools to further promote lipid production in other oleaginous microalgae.

## Results

### Generation of FAX1/FAX2/ABCA2-OE of Chlamydomonas

**Fig. 1:**
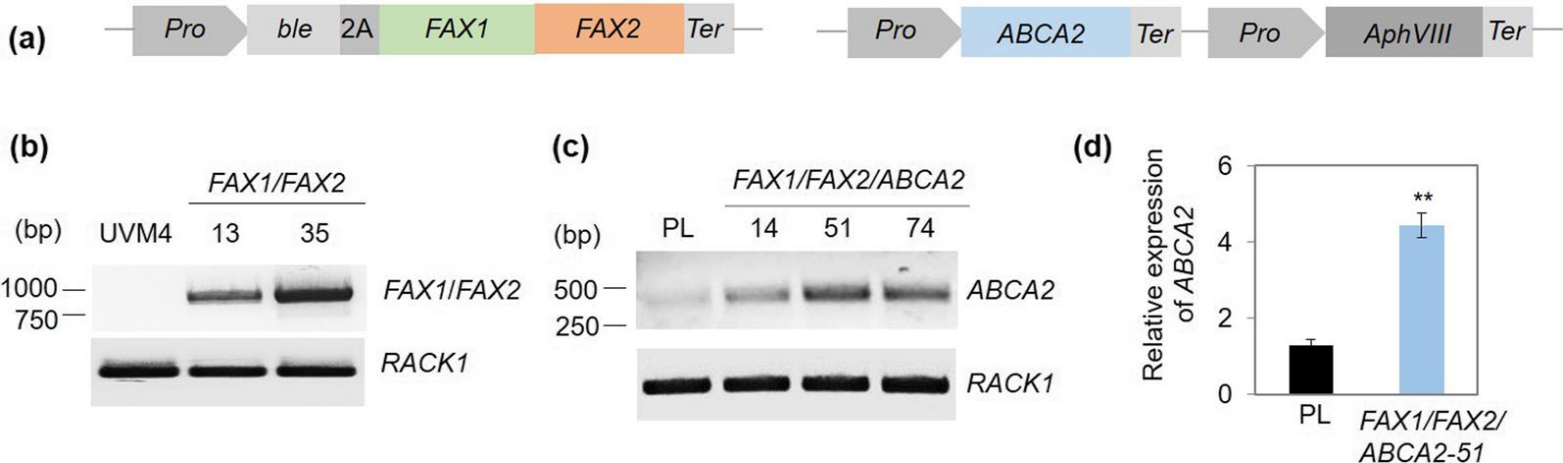
Overexpression of *FAX1*/*FAX2* and *ABCA2* in *Chlamydomonas*. **a** Schematic representation of the transformation vectors for generation of *FAX1/FAX2* (left panel), and *FAX1/FAX2/ABCA2* (right panel) overexpressing strains. *Pro*, Hsp70A/RbcS2 promoter; 2A, FMDV 2A peptide; *Ter*, Chlamydomonas RbcS2 terminator. **b**, **c** Reverse-transcription PCR (RT-PCR) analysis of the expression of *FAX1/FAX2*, and *ABCA2* in the transformants. PL, parental line (*FAX1-FAX2-35*). Housekeeping gene used is *RACK1* (*Cre06.g278222*, receptor of activated protein kinase C 1 initially described as CBLP). The PCR products amplified with the primers (FAX1-F/FAX2-R and ABCA2-F1/ABCA2-R1) at 28 cycles were shown, respectively. This is a representative of three independent experiments. **d** Quantitative RT-PCR analysis of the expression level of *ABCA2* in *FAX1/FAX2/ABCA2* transformant. The qRT-PCR results were normalized by the level of *RACK1* expression. PL, parental line (*FAX1-FAX2-35*). Error bars represent standard errors based on three biological replicates (independent cultures) with three technical replicates each. Asterisks indicate statistically significant changes compared to the parental line *FAX1-FAX2-35* by paired-sample Student’s *t* test (**P ≤ 0.01)

As a strategy to further enhance the oil content, we aimed to over-express the two FAX proteins responsible for fatty acid (FA) export from chloroplasts, and ABCA2, which helps deliver exported FAs to the ER, where the TAG synthesis mainly occurs. The *Chlamydomonas* UVM4 strain was transformed by electroporation with the pChlamy4-FAX1/FAX2 plasmid, where the DNA sequences of *FAX1* (Cre10.g421750) and *FAX2* (Cre08.g366000) were tandemly fused and driven by the hybrid promoter (Hsp70A-RbcS2) (Fig. 1a). Transformants were selected on TAP/agar plates supplemented with 15 mg/L zeocin. Zeocin-resistant clones were screened by PCR to verify the integration of pChlamy4-FAX1/FAX2 using specific primers to amplify the full-length DNA sequences of *FAX1* and *FAX2*. A band corresponding to the expected size of 2092 bp was detected in the positive transformants (Additional file 1: Fig. S1a), which confirms the integration of DNA sequences of *FAX1* and *FAX2* into the genome of *Chlamydomonas*. The PCR-positive clones (*FAX1/FAX2-13* and *-35*) were chosen for further analysis by RT-PCR using specific oligonucleotides to amplify the full-length cDNA sequences of *FAX1* and *FAX2*. The predicted size of 996 bp of *FAX1* and *FAX2* cDNA was detected in the *FAX1/FAX2-13* and *-35* clones but not in the parental strain UVM4 (Fig. 1b), which further confirmed that *FAX1* and *FAX2* were expressed in transformants.

To generate *FAX1/FAX2/ABCA2*-OE of *Chlamydomonas*, the *FAX1/FAX2-35* clone was used as the parental strain, where *FAX1/FAX2* was highly expressed. ER-located ABCA2-encoding cDNA (Cre14.g613950) was subcloned into the pOpt-Clover-Paro vector to make the pOpt-ABCA2-Paro plasmid (Fig. 1a). After the transformation, the transformants were screened on agar plates containing 15 mg/L zeocin and 20 mg/L paromomycin. The insertion of the pOpt-ABCA2-Paro construct into the genome of selected colonies was confirmed by PCR (Additional file 1: Fig. S1b, c). Furthermore, the results show that the expression levels of *ABCA2* in three representative *FAX1/FAX2/ABCA2*-OE lines significantly increased compared with those in the untransformed parental line (Fig. 1c). For example, the expression level of *ABCA2* increased by up to 3.4-fold in *FAX1/FAX2/ABCA2-51* compared to its parental strain UVM4, as analysed by qRT-PCR (Fig. 1d).

### FAX1/FAX2/ABCA2-OE exhibit increased TAG accumulation

**Fig. 2:**
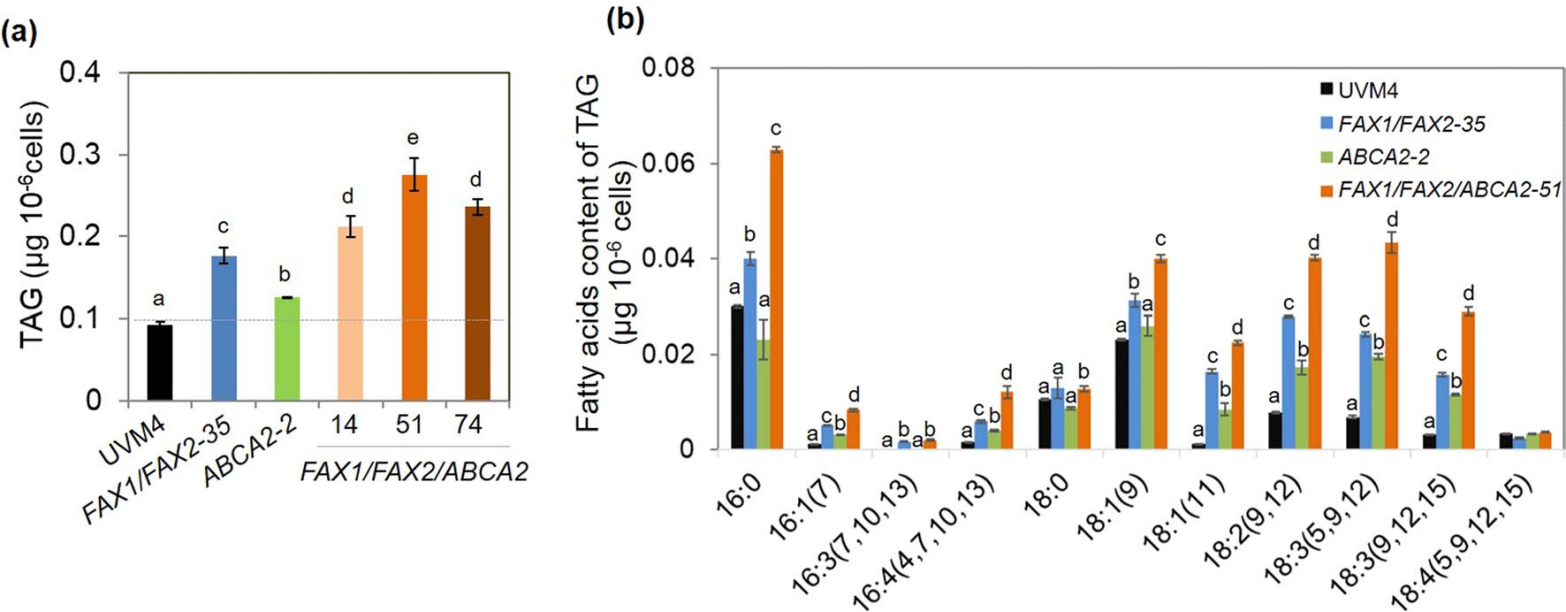
Levels of TAG and profile of fatty acids content of TAG in the transformants at exponential phase (day 2) under standard growth conditions. **a** TAG content, **b** fatty acids content of TAG. The distinct letters labelled indicate the statistically significant difference by Tukey’s honestly significant difference (HSD) test

Changes in oil content in the triple over-expressor (*FAX1/FAX2/ABCA2*-OE) with the single over-expressor for *ABCA2*-OE [16], double over-expressor (*FAX1/FAX2*-OE), and untransformed parental strain (UVM4) were analysed here using thin-layer chromatography (TLC) coupled to GC–MS analysis. The strains were grown side by side under identical growth conditions for each experiment. Under standard growth conditions, we observed that *FAX1/FAX2-35* and *ABCA2-2* showed 91% and 36% more TAG than UVM4, while *FAX1/FAX2/ABCA2-OE* exhibited up to twofold more TAG than UVM4. Compared to the *FAX1/FAX2-35* strain, there was 56% more TAG in *FAX1/FAX2/ABCA2-51* when *ABCA2* was further over-expressed (Fig. 2a). Under nitrogen (N)-depleted conditions, all three FA transporter-OE also showed increased TAG content compared with UVM4 (Additional file 1: Fig. S2a). Interestingly, the fold change in the increase in TAG contents under standard growth conditions in FA transporter-OE was comparable or even higher than that under N-depleted conditions (Fig. 2a; Additional file 1: Fig. S2a). Among the three independent strains of *FAX1/FAX2/ABCA2*-OE, *FAX1/FAX2/ABCA2-51* was selected for further study due to its higher expression level of *ABCA2* (Fig. 1c) and higher TAG content than the other strains under standard growth and N-depleted conditions (Fig. 2a; Additional file 1: Fig. S2a).

The analysis of the individual FA contents of TAG reveals that almost all monounsaturated FA and PUFA contents increased in all three FA transporter-OE compared with UVM4 (Fig. 2b). Furthermore, the results show that *FAX1/FAX2-35* exhibited some differences from *ABCA2-2* in the augmentation of FA contents in TAG. In *FAX1/FAX2-35*, the contents of 16:0, 16:3 (7,10,13) and 18:1 (9) increased compared with UVM4, but they did not change in *ABCA2-2*. Noticeably, all FA contents of TAG significantly increased in *FAX1/FAX2/ABCA2-51* compared with individual FA transporter-OE and UVM4, except 18:4 (5,9,12,15), which was unchanged (Fig. 2b). Taken together, these results indicate that the increased expression of *FAX1/FAX2* and *ABCA2* individually enhanced the oil accumulation, and this effect was further enhanced by simultaneously expressing the three genes.

### FAX1/FAX2/ABCA2 over-expression increased the total fatty acids

**Fig. 3:**
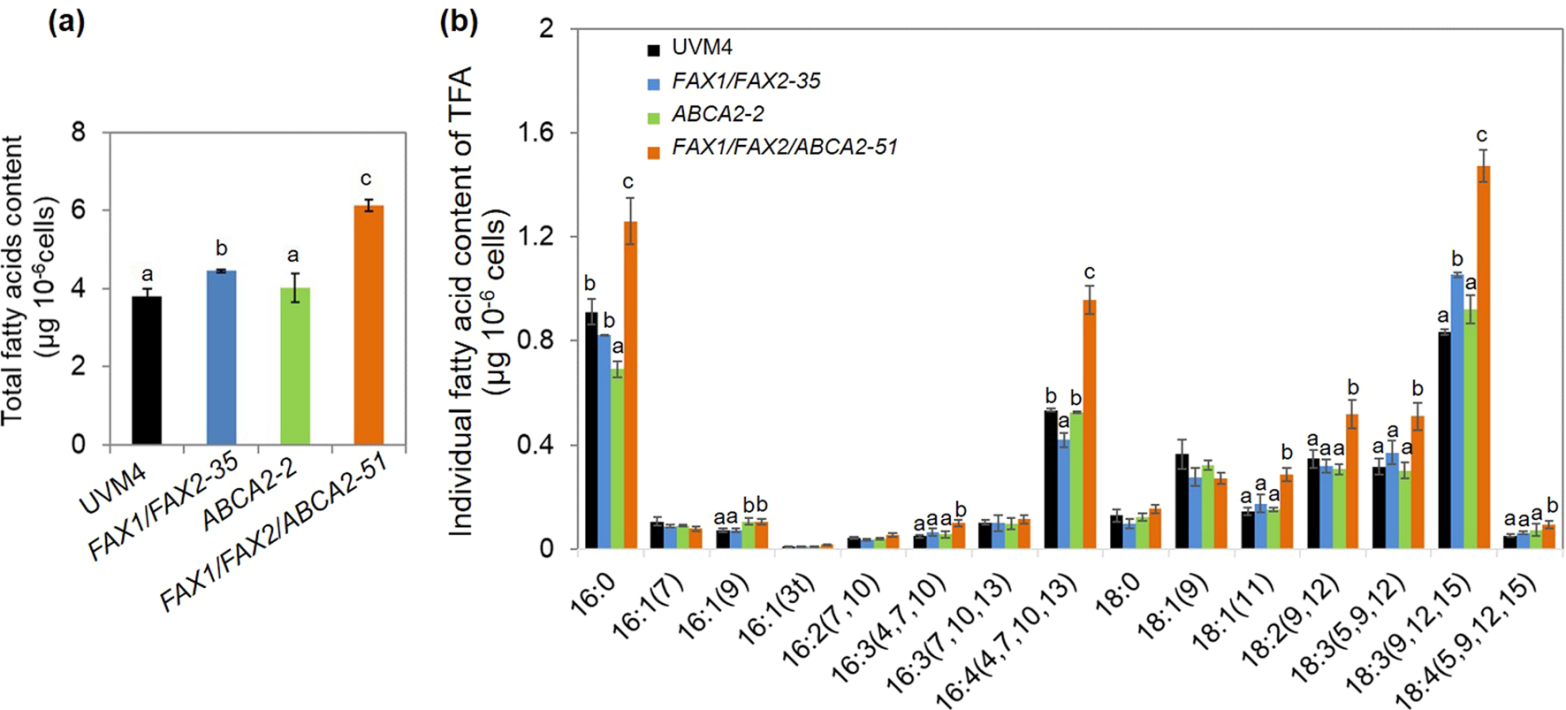
Total and individual fatty acids content in the transformants of *FAX1*/*FAX2* and *ABCA2* at exponential phase (day 2) under standard growth conditions. **a** Total fatty acids content. **b** Profile of individual fatty acid content in total fatty acids (TFA). The distinct letters labelled indicate the statistically significant difference by Tukey’s HSD test

We further examined the influence of *FAX1/FAX2/ABCA2* over-expression on the FA content under standard growth conditions. Compared with the untransformed parental line UVM4, the total FA contents significantly increased in *FAX1/FAX2-35* and *FAX1/FAX2/ABCA2-51* but were not different from *ABCA2-2*. However, when *ABCA2* was over-expressed in *FAX1/FAX2-35*, there were 38% and 61% more total FA in *FAX1/FAX2/ABCA2-51* than in the parental strains *FAX1/FAX2-35* and UVM4, respectively (Fig. 3a). The increase of total FA contents was also found in other *FAX1/FAX2/ABCA2-*OE, such as *FAX1/FAX2/ABCA2-14* and *FAX1/FAX2/ABCA2-74* (Additional file 1: Fig. S3). The individual FA content analysis shows that the contents of major FA (e.g., 16:0, 16:4 and 18:3) increased in *FAX1/FAX2/ABCA2-51* compared with the control strains (UVM4, *FAX1/FAX2-35* and *ABCA2-2*) (Fig. 3b). However, the individual FA content did not greatly change in either *FAX1/FAX2*-OE or *ABCA2*-OE compared with UVM4. In *FAX1/FAX2-35*, only 18:3 (9,12,15) increased, and 16:4 (4, 7, 10, 13) decreased, but other FA contents did not significantly change. In *ABCA2-2*, 16:1 (9) increased, but 16:0 was reduced (Fig. 3b). Additionally, the effect of these FA transporters on FA content was explored under N-depleted conditions. All total FA contents significantly increased in *FAX1/FAX2-35*, *ABCA2-2* and *FAX1/FAX2/ABCA2-51* compared with UVM4 (Additional file 1: Fig. S2b). For example, the contents of saturated FAs (e.g., 16:0 and 18:0) more significantly increased in both *FAX1/FAX2-35* and *ABCA2-2* than in UVM4. Noticeably, all FA levels were increased in *FAX1/FAX2/ABCA2-51* compared with the control strains under N-depleted conditions (Additional file 1: Fig. S2c). Therefore, our results indicate that the increased expression of *FAX1/FAX2/ABCA2* is likely to balance different substrate preferences towards fatty acyls among these different FA transporters and positively contributes to the accumulation of saturated and unsaturated FAs.

### Major membrane lipid levels were also altered in FAX1/FAX2/ABCA2-OE

**Fig. 4:**
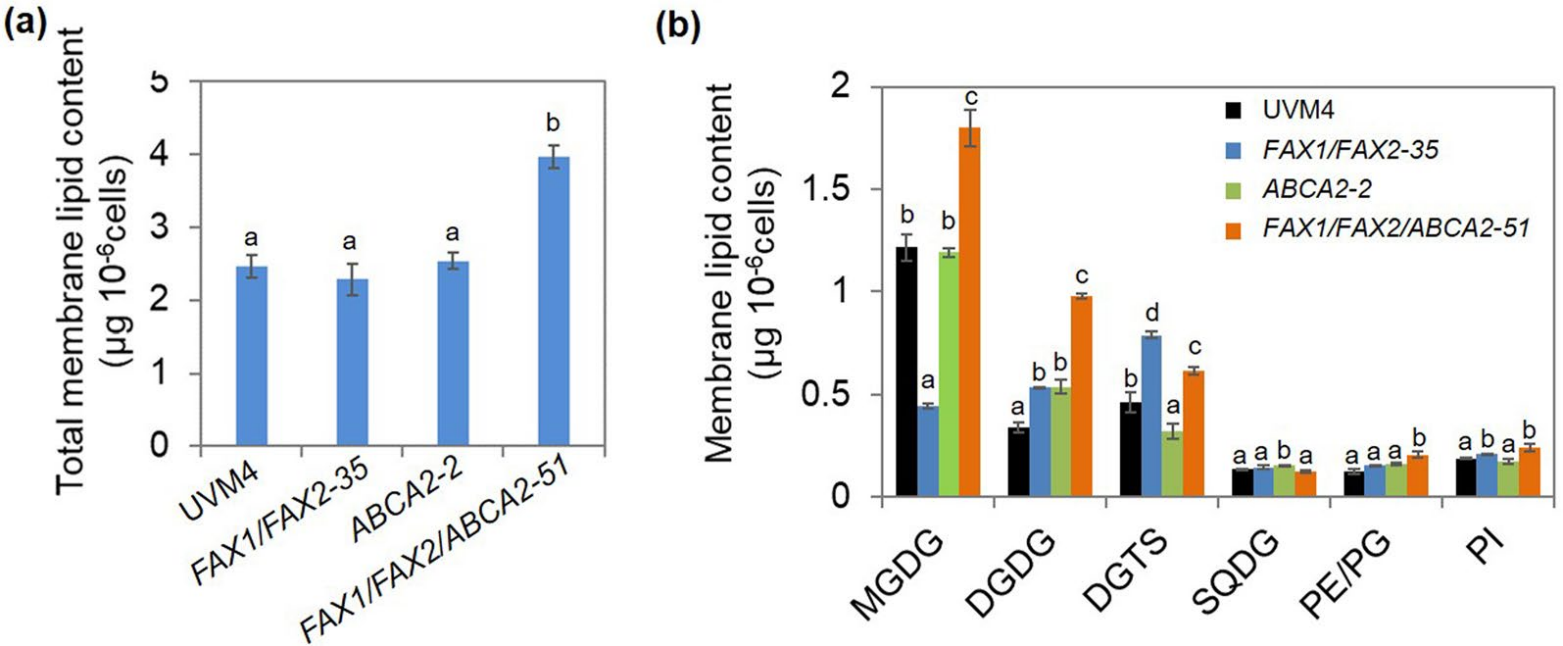
Alterations of membrane glycerolipid levels in the transformants of *FAX1*/*FAX2* and *ABCA2* at exponential phase (day 2) under standard growth conditions. **a** Total membrane lipid content. **b** Profile of individual membrane component contents. The total membrane lipid content referred to the total amount of major galactolipids (MGDG, DGDG and SQDG) and phospholipids (PE, PG and PI), and betaine lipid DGTS. MGDG, monogalactosyldiacylglycerol; DGDG, digalactosyldiacylglycerol; DGTS, diacylglycerol-*N*,*N*,*N*-trimethylhomoserine; SQDG, sulfoquinovosyldiacylglycerol; PG, phosphatidylglycerol; PE, phosphatidylethanolamine; PI, phosphatidylinositol. The distinct letters labelled indicate the statistically significant difference by Tukey’s HSD test

To determine whether the increases in TAG and total FA in *FAX1/FAX2/ABCA2*-OE are accompanied by changes in polar lipids, the major membrane glycerolipids were analysed, including MGDG (monogalactosyldiacylglycerol), DGDG (digalactosyldiacylglycerol) and DGTS (diacylglyceryltrimethylhomoserine). The results show that the total membrane lipid content increased by 61% in *FAX1/FAX2/ABCA2-51* compared with the untransformed parental strain UVM4, while no changes were found in *FAX1/FAX2-35* and *ABCA2*-*2* (Fig. 4a). In *FAX1/FAX2-35*, the amount of the major thylakoid membrane lipid MGDG decreased by 48%, while the second most abundant thylakoid membrane lipid DGDG and the major extraplastidic lipid DGTS both prominently increased compared with UVM4 (Fig. 4b). These results are consistent with previous reports on these two genes (*FAX1* and *FAX2*) individually expressed in *Chlamydomonas* [17]. In *ABCA2-2*, the amount of DGDG significantly increased, while the content of DGTS decreased, and that of MGDG did not change compared with UVM4 (Fig. 4b). However, when *FAX1*, *FAX2* and *ABCA2* were co-expressed, all major membrane lipid levels in *FAX1/FAX2/ABCA2-OE* increased compared with those in UVM4, except that the SQDG (sulfoquinovosyldiacylglycerol) amount was unaltered (Fig. 4b; Additional file 1: Fig. S4). For example, in *FAX1/FAX2/ABCA2-51*, the contents of MGDG, DGDG and DGTS were 48%, 188% and 34% higher than those in UVM4, respectively (Fig. 4b). Furthermore, the contents of unsaturated FAs [16:3 (4,7,10), 16:4 (4,7,10,13), 18:2 (9,12) and 18:3 (9,12,15)] of MGDG were significantly higher in *FAX1/FAX2/ABCA2-51* than in UVM4 (Additional file 1: Fig. S5a); for example, both major FAs of MGDG [16:4 (4,7,10,13) and 18:3 (9,12,15)] increased by more than 60%. The saturated FA (e.g., 16:0) and almost all detected unsaturated FAs in DGDG, such as 16:2 (7,10), 16:3 (7,10,13), 18:2 (9,12), and 18:3 (9,12,15) notably increased (Additional file 1: Fig. S5b). In particular, the contents of the major FAs of DGDG [18:2 (9,12) and 18:3 (9,12,15)] increased by 177% and 128% in *FAX1/FAX2/ABCA2*-51 compared with UVM4, respectively. In the FA profile of DGTS, the contents of saturated FA 16:0 and almost all unsaturated FA also increased in *FAX1/FAX2/ABCA2*-51 compared with UVM4 (Additional file 1: Fig. S5c). For example, the major FA of DGTS, 18:3 (5,9,12), increased by 73%. These results indicate that the phenotype of higher lipid contents observed in *FAX1/FAX2/ABCA2*-OE is probably mainly due to an increase in the de novo biosynthesis of FA, and the higher TAG level in this strain may be accompanied by de novo-synthesized membrane lipids, including MGDG, DGDG and DGTS, under normal growth conditions.

### Analysis of the contribution source to accumulate total FA, TAG and membrane lipids in FAX1/FAX2/ABCA2-OE

To further elucidate the major contributors to the lipid accumulation, the contribution ratio of TAG and membrane lipids (PL) to total FA accumulation increased in *FAX1/FAX2/ABCA2-OE* relative to the parental strain UVM4 were firstly examined. The results showed that the contribution ratio of PL to total FA accumulation in *FAX1/FAX2/ABCA2-51* was sevenfold than that of TAG relative to UVM4 (Additional file 1: Fig. S6a), which indicates that PL is the major contributor to total FA accumulation in *FAX1/FAX2/ABCA2-OE*. Furthermore, the main contributors to PL accumulation were MGDG, DGDG and DGTS in *FAX1/FAX2/ABCA2-51* with contribution ratios of 39%, 43% and 10% in relative to UVM4, respectively (Additional file 1: Fig. S6b). Therefore, the biosynthesis of these three membrane lipids was remarkably enhanced in the over-expressed *FAX1/FAX2/ABCA2-51*, especially the thylakoid membrane lipids MGDG and DGDG. We also found that the PUFA levels of total FA, TAG and the major PL were significantly higher in *FAX1/FAX2/ABCA2-51* than in UVM4 (Additional file 1: Fig. S6c). Then, we analysed the contribution ratio of the major PUFA to total FA accumulation, and the results showed that the increased ratio of 18:3 (9,12,15) in *FAX1/FAX2/ABCA2-51* was the most prominent, followed by 16:4 (4,7,10,13) relative to UVM4 (Additional file 1: Fig. S6d). The contribution ratios of the major FAs 18:3 (9,12,15) and 16:4 (4,7,10,13) to MGDG accumulation were more than 40%, and that of 18:3 (5,9,12) for DGTS was more than 50% in *FAX1/FAX2/ABCA2-51* relative to UVM4 (Additional file 1: Fig. S6e). For DGDG accumulation, the contribution ratios of major FA 18:2 (9,12) and 18:3 (9,12,15) were also significantly higher in *FAX1/FAX2/ABCA2-51* than in UVM4, although they were less than those of the major FAs in MGDG and DGDG (Additional file 1: Fig. S6e)*.* Additionally, the total accumulation contributions of major PUFAs, e.g., 16:4 (4,7,10,13), 18:3 (9,12,15) and 18:3 (5,9,12), to the total FA and TAG accumulation in *FAX1*/*FAX2*/*ABCA2-51* were 54% and 40% higher than UVM4, respectively (Additional file 1: Fig. S6d, f). Overall, the contents of PUFA, which serve as the major contributors to total FA, TAG and the major membrane lipids in *FAX1*/*FAX2*/*ABCA2-*OE, significantly increased when fatty acid and ABC transporters were co-expressed.

### Expression levels of genes in the TAG biosynthesis and membrane lipid turnover in FAX1/FAX2/ABCA2-OE

**Fig. 5:**
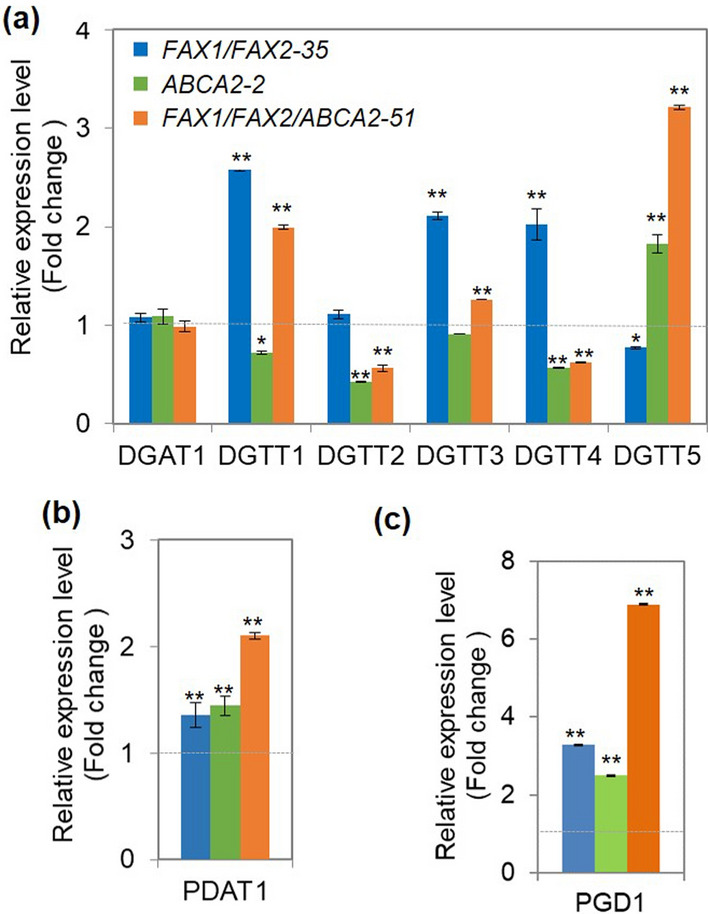
qRT-PCR analysis of the expression of key genes in the TAG biosynthesis and membrane lipid turnover in the transformants at exponential phase (day 2) under standard growth conditions. The relative expression level of diacylglycerol acyltransferases (DGAT) (**a**), phospholipid:diacylglycerol acyltransferase 1 (PDAT1) (**b**), and plastid galactoglycerolipid degradation 1 (PGD1) (**c**). The housekeeping gene *RACK1* (*Cre06.g278222*) was used as the internal standard. Values are the relative expression level compared with parental line UVM4. Error bars represent standard deviations based on three biological replicates (independent cultures) with three technical replicates each. Asterisks indicate statistically significant changes compared to the parental line UVM4 by paired-sample Student’s *t* test (*P ≤ 0.05; **P ≤ 0.01)

To gain insights into the mechanisms of enhancing lipid accumulation when *FAX1*, *FAX2*, and *ABCA2* were co-expressed, we first analysed the transcript levels of key enzymes for the TAG biosynthesis, diacylglycerol acyltransferases (DGAT) and phospholipid:diacylglycerol acyltransferase (PDAT), which are directly involved in the acylation of diacylglycerol and transacylation of phospholipids into TAG. The results showed that the expression of *DGAT1* (type-I DGAT) was unaltered in the three FA transporter-OE. However, type-II DGAT showed differential expression levels in the three FA transporter-OE compared with UVM4 (Fig. 5a). In *FAX1/FAX2-35*, the expression of *DGTT1*, *DGTT3* and *DGTT4* was significantly upregulated (> 2.0-fold), while *DGTT5* was slightly downregulated compared with UVM4 (Fig. 5a). In *ABCA2-2*, the expression of *DGTT5* was upregulated by 1.8-fold, while that of *DGTT1*, *DGTT2* and *DGTT4* was slightly downregulated compared with UVM4 (Fig. 5a). When *FAX1*, *FAX2*, and *ABCA2* were co-expressed, the expression levels of *DGTT1*, *DGTT3* and *DGTT5* were upregulated 2.0-fold, 1.3-fold and 3.2-fold than UVM4, respectively, compared with UVM4. However, the expression of *DGTT2* and *DGTT4* was downregulated in *FAX1/FAX2/ABCA2-51* compared with UVM4 (Fig. 5a). Interestingly, the expression level of *PDAT1* was upregulated in all three FA transporter-OE compared to UVM4 (Fig. 5b). Moreover, the expression level of MGDG-specific lipase (*PGD1*) was significantly upregulated (> twofold) in all three FA transporter-OE, especially that in *FAX1/FAX2/ABCA2-51* (up to sevenfold) (Fig. 5c). The significantly upregulated expression of *PDAT1* and *PGD1* in *FAX1*/*FAX2*/*ABCA2-*OE, *FAX1*/*FAX2*- and *ABCA2-*OE revealed the vital roles of membrane lipid remodelling, especially MGDG, in the TAG biosynthesis. Overall, *FAX1/FAX2/ABCA2-OE* showed significantly higher expression of *DGAT* (*DGTT1*, *DGTT3* and *DGTT5*), *PDAT* (*PDAT1*) and MGDG-specific lipase (*PGD1*) than UVM4, which was likely to account for the obvious accumulation of TAG in the *FAX1/FAX2/ABCA2-OE* strain.

### Physiological consequence of FAX1/FAX2/ABCA2-OE

**Fig. 6:**
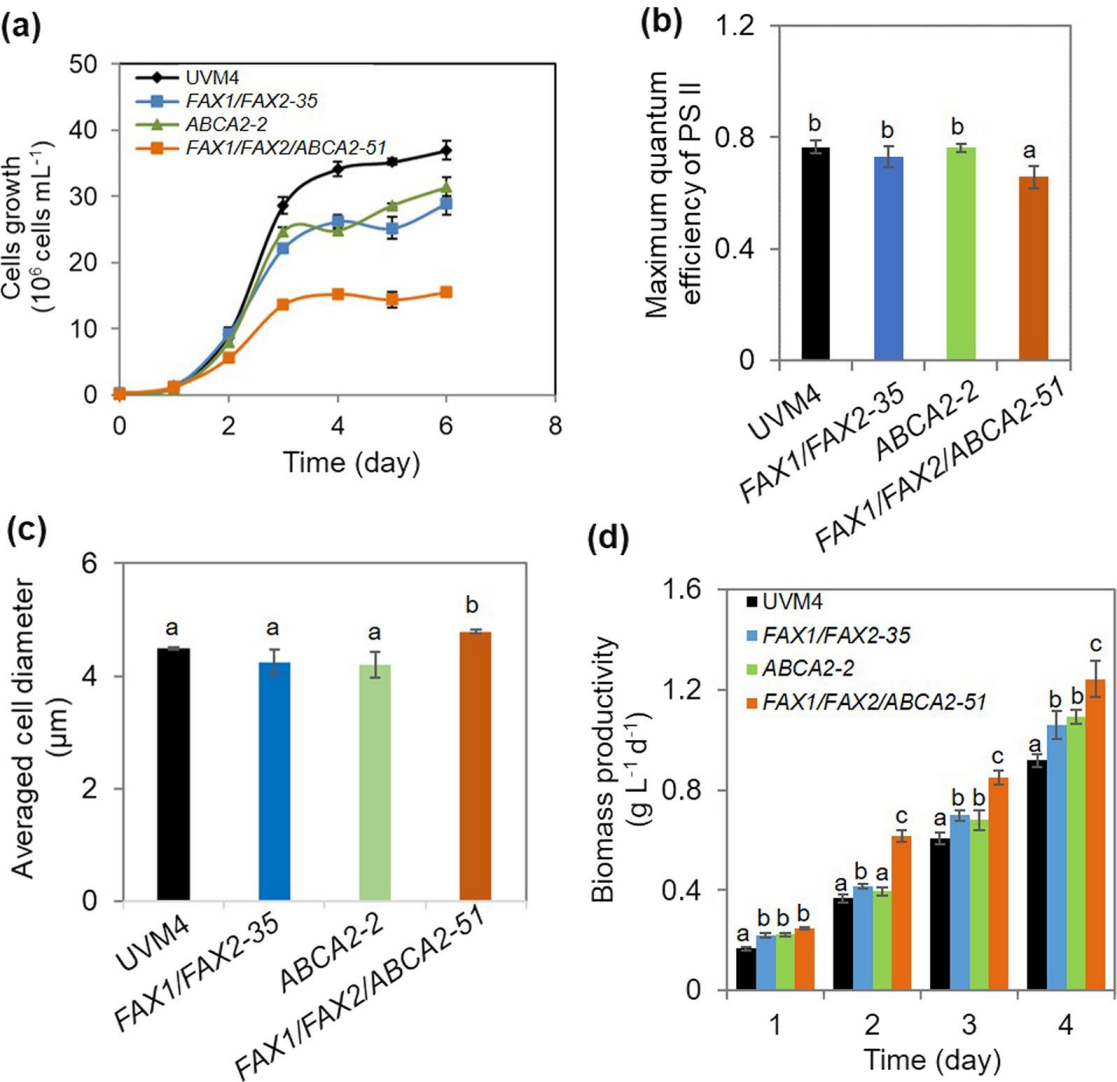
Phenotypic changes of the transformants under standard growth conditions. **a** Cell growth kinetics. **b** The maximum quantum efficiency (*F*_v_*/F*_m_) of photosystem II (PS II) **c** Averaged cell diameter. **d** Biomass productivity. In **b** and **c**, the cells were harvested at exponential phase (day 2) under standard growth conditions. The distinct letters labelled indicate the statistically significant difference by Tukey’s HSD test

The physiological effect on the cells was also examined, when *FAX1*, *FAX2*, and *ABCA2* were co-expressed. The results showed that the cell growth rate of *FAX1/FAX2/ABCA2-51* was significantly slower than that of the untransformed parental line UVM4, and the over-expression of either *FAX1/FAX2* or *ABCA2* only delayed growth in the stationary phase under standard growth conditions (Fig. 6a). The slow cell growth of the triple over-expressor is consistent with a lowered maximum quantum efficiency (*F*_v_*/F*_m_) of PS II compared with all control strains (UVM4, *FAX1/FAX2-35* and *ABCA2-2*) (Fig. 6b). However, *FAX1/FAX2/ABCA2-51* had bigger cell size and biomass productivity than all control strains (Fig. 6c, d). This result is consistent with a previous report that when *FAX1* or *ABCA2* homologous genes (*AtFAX1* or *AtABCA9*) were over-expressed in *Arabidopsis thaliana*, the mutants showed an increase in dry weight compared with wild type [18, 19]. Our results suggested that the increased expression of *FAX1/FAX2/ABCA2* contributes to the increase in cell size and biomass productivity of microalgae.

## Discussion

**Fig. 7:**
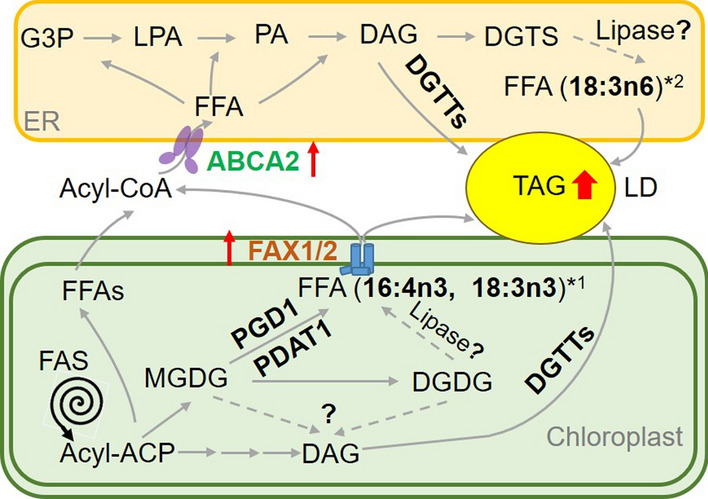
Hypothetical model explaining adjustment of lipid metabolism under standard growth conditions when *FAX1*, *FAX2* and *ABCA2* were co-expressed in *Chlamydomonas*. ABCA2, ATP-binding cassette transporter subfamily A2; ACP, acyl carrier protein; DAG, diacylglycerol; DGTS, diacylglyceryltrimethylhomoserine; DGTTs, type II diacylglycerol acyltransferases; DGDG, digalactosyldiacylglycerol; ER, endoplasmic reticulum; FAX1/2, fatty acids exporters 1 and 2; FAS, fatty acid synthase; FFA, free fatty acids; G3P, glycerol-3-phosphate; LD, Lipid droplet; LPA, lysophosphatidic acid; MGDG, monogalactosyldiacylglycerol; PA, phosphatidic acid; PGD1, plastid galactoglycerolipid degradation 1; PDAT1, phospholipid:diacylglycerolacyltransferase 1; TAG, triacylglycerol. The 16:4n3 [16:4(4,7,10,13)]^*1^, 18:3n3 [18:3(9,12,15)] and 18:3n6 [18:3(5,9,12)]^*2^ derived from membrane lipids were highlighted in bold

Chloroplast- and ER-localized FA transporters were reported to play important roles in TAG biosynthesis in *Chlamydomonas reinhardtii* [16, 17]. However, whether FA transporters synergistically contribute to lipid accumulation in microalgae is unknown. In this study, we found that under standard growth conditions, *FAX1/FAX2/ABCA2*-OE showed greatly more TAG and total amounts of major PUFA in TAG than the untransformed parental line (Fig. 2). Moreover, our results suggested that the contents of total FA and major membrane lipids also significantly increased in *FAX1/FAX2/ABCA2*-OE compared with the control strains (Figs. 3, 4). Additionally, the total accumulation contribution ratios of major PUFA to total FA and TAG synthesis in *FAX1*/*FAX2*/*ABCA2-*OE were both higher than UVM4 (Additional file 1: Fig. S6). Consistently, the expression of key genes in the TAG synthesis and major thylakoid membrane turnover was significantly upregulated in *FAX1*/*FAX2*/*ABCA2-*OE (Fig. 5). These results indicated that the simultaneously increased expression of *FAX1*, *FAX2* and *ABCA2* has an additive effect on enhancing the TAG and membrane lipid accumulation, and prominently accelerates the PUFA remobilization from membrane lipids to TAG by fine-tuning the key genes in lipid metabolism (Fig. 7).

In this study, *ABCA2-OE* (*ABCA2-2*) was generated by overexpressing of *ABCA2* cDNA and characterized as we previously described [16]. To compare with the same single mutant (*ABCA2-2*), we generated *FAX1/FAX2/ABCA2-OE* using *ABCA2* cDNA. Recently, it was reported that intron-containing transgenes mediate efficient recombinant gene expression in *Chlamydomonas* [20, 21]. We used the genomic DNA of *FAX1/FAX2* for the generation of *FAX1/FAX2/ABCA2-OE*, considering that native regulatory elements (introns) might facilitate the expression of nuclear transgenes. It is worth testing whether the expression levels of *FAX1/FAX2/ABCA2* can be enhanced using a synthetic intron spread strategy in the future.

The accumulation of TAG is usually accompanied by the degradation of the membrane lipids in microalgae [10, 22, 23]. Interestingly, our results showed that the content of total membrane lipids significantly increased in the *FAX1/FAX2/ABCA2*-OE strain compared with the untransformed parental strain, while no changes were observed in the *FAX1/FAX2*-OE and *ABCA2*-OE strains (Fig. 4a). In *FAX1/FAX2-35*, the major lipid component of the thylakoid membrane, such as MGDG, significantly decreased, while DGDG and the major extraplastidic lipids such as DGTS increased compared with the untransformed parental strain (Fig. 4b), which is in consistent with previous reports on these two genes (*FAX1* or *FAX2*) being individually expressed in *Chlamydomonas* [17]. In *ABCA2-2*, the amount of DGDG significantly increased, while MGDG was not changed compared with the untransformed parental strain (Fig. 4b). However, when the expression of *FAX1*, *FAX2*, and *ABCA2* was synergistically increased, all major membrane lipids in *FAX1/FAX2/ABCA2-51* increased compared with the untransformed parental line and individual FA transporter-OE (Fig. 4b). The contribution ratio of polar lipids to the accumulation of total FA in *FAX1/FAX2/ABCA2-51* was up to 65%, while that of TAG was only 9%, compared with the parental strain UVM4 (Additional file 1: Fig. S6a). Noticeably, the biosynthesis of membrane lipids was remarkably enhanced in *FAX1/FAX2/ABCA2-51*, especially the plastidial photosynthetic membrane lipids MGDG and DGDG (Fig. 4b). Subsequently, all ratios of bilayer-forming lipid DGDG to nonbilayer-forming lipid MGDG in three FA transporter-OE increased (> twofold) compared with UVM4 (calculated from Fig. 4b), which indicates that the elevated membrane adaptations are similar to heat-stressed *Chlamydomonas* and higher plants [24]. Therefore, head group exchanges of glycerolipids can also be a mechanism that *Chlamydomonas* employs to adapt the over-expression of these FA transporters.

In microalgae, acyl-ACPs are generated by FA synthesis and cleaved by thioesterases (TEs) to form free FAs in the chloroplast. Free FAs are exported from the plastid to the ER, and esterified to acyl-CoAs to form TAG [10, 25]. The key enzymes involved in fatty acid synthesis (e.g., ACCase) are feedback-inhibited by long-chain fatty acyl-ACPs, and the expression of endogenous acyl-ACP TEs can relieve this inhibitory mechanism in microalgae [26]. For example, the over-expression of endogenous *TEs* in the diatom *Phaeodactylum tricornutum* enhanced its total FA content by 72% [27]. Moreover, heterologous over-expression of *TEs* from *Dunaliella tertiolecta* increased both neutral lipid and total FA content in *Chlamydomonas* [26]. Our results showed that both TAG and total FA also increased in *FAX1/FAX2/ABCA2-OE*. One of the possible reasons may partially be due to the TE-related deregulation in lipid metabolism, which must be studied in the future studies.

In terms of polyunsaturation, either total fatty acyls or main membrane lipids, i.e., MGDG, DGDG and DGTS, exhibited prominently incremental abundances of polyunsaturated fatty acyls in *FAX1/FAX2/ABCA2-51* (Additional file 1: Fig. S6c). Meanwhile, the PUFAs attached with more than two double bonds [e.g., 16:4 (4,7,10,13), 18:3 (5,9,12) and 18:3 (9,12,15)], prominently accumulated in *FAX1/FAX2/ABCA2-51* relative to UVM4 (Fig. 2b). These PUFA mainly reside in membrane lipids, and their assembly involves elongation and desaturation, which also depend on carrier membrane lipids [28, 29]. The main contributor to MGDG augmentation was its primary fatty acids, 16:4 (4,7,10,13) and 18:3 (9,12,15), and their contribution ratios both increased (up to 44%) relative to UVM4 (Additional file 1: Fig. S6e), which indicates the vital role of the main molecule 18:3 (9,12,15)/16:4 (4,7,10,13) in the MGDG of *FAX1/FAX2/ABCA2-OE*. In the second most abundant photosynthetic galactolipid, DGDG, the contribution ratio of 18:3 (9,12,15) was 18% and that of 18:2 (9,12) was 13% in *FAX1*/*FAX2*/*ABCA2-51* (Additional file 1: Fig. S6e). In parallel, the pivotal functional molecules of DGDG were 18:3 (9,12,15)/16:0 and 18:2 (9,12)/16:0. In contrast, the major FA of the ER-located betaine lipid DGTS is 18:3 (5,9,12) [30, 31], whose contribution ratio to DGTS formation was up to 56% in *FAX1*/*FAX2*/*ABCA2-51* (Additional file 1: Fig. S6e). The key molecule in DGTS was considered to be 18:3 (5,9,12)/16:0, whose content was found to notably increase in *FAX1*/*FAX2*/*ABCA2-51*. In this study, we observed the co-accumulation of TAG and photosynthetic membrane lipids (mainly MGDG and DGDG) in *FAX1*/*FAX2*/*ABCA2-*OE, which might be a response to over-expression of three fatty acid transporters.

The conversion of diacylglycerol into TAG is the only committed final step to TAG synthesis in plants and microalgae, which is mainly catalysed by DGAT and PDAT [29]. In *Chlamydomonas*, there is only one characterized PDAT (PDAT1) [32], but six DGATs were found [13, 32]. PDAT1 is a multi-functional enzyme with proven galactolipase function that prefers membrane lipids, particularly de novo-synthesized MGDG, as substrates to synthesize TAG in *Chlamydomonas* [32, 33]. Recently, it was reported that the less-unsaturated-PE, which serves as a transient carbon reservoir, could be used by PDAT for oil biosynthesis in *Nannochloropsis* [34]. Therefore, the increased PE/PG in our study may also be used as a PDAT1 substrate to enhance the TAG biosynthesis in *Chlamydomonas*. The *Chlamydomonas pgd1* null mutant reduced the turnover of de novo-synthesized MGDG, which decreased the TAG content [35]. In this study, significantly upregulated expression levels of *PDAT1* and *PGD1* in *FAX1*/*FAX2*/*ABCA2-*OE were found (Fig. 5b, c), which revealed their critical roles in membrane lipid remodelling, especially MGDG for TAG biosynthesis. The major FAs of MGDG, i.e., 16:4 (4,7,10,13) and 18:3 (9,12,15), are proposed to be hydrolysed by these two lipases (PDAT1 and PGD1). The fatty acyls released from MGDG may serve as the acyl pool for TAG assembly catalysed by type-II DGAT in *FAX1*/*FAX2*/*ABCA2-*OE (Fig. 7).

In microalgae, the substrates used for TAG synthesis can partially originate from the main polar lipid species, since the FA from the degraded membrane lipids can be recycled for TAG synthesis [3]. The total membrane lipid content increased (Fig. 4b), and two major PUFAs [16:4 (4,7,10,13) and 18:3 (9,12,15)] were synthesized more in the *FAX1*/*FAX2*/*ABCA2-*OE strain than in the parental strain UVM4 (Fig. 3b). These enriched PUFA in TAG are likely to originate from the turnover pathway of the newly synthesized photosynthetic membrane lipids instead of that of the nascent ones when the expression of three fatty acid transporters was simultaneously increased. Our results showed that the expression levels of key enzymes in the TAG synthesis, such as *DGTT1*, *DGTT3*, *DGTT5*, *PDAT1* and *PGD1*, in the MGDG-specific degradation were upregulated in *FAX1/FAX2/ABCA2*-*51* comparted with the parental strain UVM4 (Fig. 5). Therefore, the higher TAG content in *FAX1/FAX2/ABCA2*-OE could be due to the enhancement of de novo synthesis of TAG and remodelling of the de novo-synthesized MGDG. Based on the gene expression levels of the related enzymes in the TAG assembly, the major photosynthetic membrane lipid MGDG is likely to direct the newly formed 16:4 (4,7,10,13) and 18:3 (9,12,15) into the stored TAG in *FAX1/FAX2/ABCA2-OE* by upregulating lipases (e.g., PDAT1 and PGD1) with acyltransferases (e.g., type-II DGAT) (Fig. 7). This study may present a novel TAG assembly pathway when FA and ABC transporters are co-expressed in *Chlamydomonas* under standard growth conditions.

The FAX1 specificity in *Arabidopsis thaliana* is significantly higher for palmitic acid than for stearic and oleic acid [19]. We also found that over-expression of two different types of transporters has different effects on FA components. Under standard growth conditions, the total FA levels increased in *FAX1/FAX2*-OE, which was mainly due to the increase in 18:3 (9,12,15); however, the total FA in *ABCA2*-OE did not change (Fig. 3). *ABCA2* homologous gene (*ABCA9*)-overexpressing plants also showed no significant differences in FA composition [18]. In this study, when *ABCA2* was over-expressed in *FAX1/FAX2*-OE, there were 38% and 61% more total FA in *FAX1/FAX2/ABCA2-51* than in the parental strains *FAX1/FAX2-35* and UVM4, respectively. Moreover, almost all FA components increased in *FAX1/FAX2/ABCA2*-*51* comparted with the untransformed parental line UVM4. In particular, the contents of 16:0, 16:4 (4,7,10,13) and 18:3 (9,12,15) significantly increased in *FAX1/FAX2/ABCA2*-*51* (Fig. 3). These results indicated that these two types of FA transporters may have different FA preferences, and a simultaneously increased expression of these transporters can balance the differences and increase the FA content. Under N-depleted conditions, the TAG, total FA and most FA component contents increased in *FAX1/FAX2/ABCA2*-OE (Additional file 1: Fig. 2). These results also suggested that the increased expression of *FAX1/FAX2*/*ABCA2* shows an additive effect on enhancing the total FA and FA component contents, in addition to the TAG and membrane lipid contents under both standard growth and N-depleted conditions.

The maximum quantum efficiency (*F*_v_*/F*_*m*_) of photosystem (PS) II was negatively correlated with lipid contents in microalgae [31, 36, 37]. Our results showed that both *FAX1/FAX2*-OE and *ABCA2*-OE showed no differences in *F*_v_*/F*_m_ compared with the untransformed parental strain UVM4. However, when the expression of *FAX1*, *FAX2* and *ABCA2* was synergistically increased, *FAX1/FAX2/ABCA2*-OE exhibited lower *F*_v_*/F*_m_ than the control lines. The relatively lower *F*_v_*/F*_m_ can cause the higher lipid phenotype in *FAX1/FAX2/ABCA2*-OE. The effect of the FA distribution and acceleration of FA transport from the chloroplast to the ER may impact the photosynthesis metabolism and inhibit growth in *Chlamydomonas*. In the microalgae *Synechococcus elongatus*, PS II is more readily photodamaged when intracellular free FA overaccumulates [38]. Therefore, the synergetic over-expression of these FA transporters may affect the intracellular and extracellular free FA levels, which may reduce the PS II activity and retard cell growth. If so, the rescue of growth inhibition by the free FA exporter should also be considered in future studies. Although the cell growth rate of *FAX1/FAX2/ABCA2*-OE was adversely affected, the biomass productivity was increased (Fig. 6a, d). The higher biomass yields may be due to bigger cells and more lipid accumulation. The protein homologues of FAX also exist in other microalgae, such *Chlorella variabilis*, *Chromochloris zofingiensis* and *Botryococcus braunii* [17] (https://phytozome-next.jgi.doe.gov/). Therefore, the engineering strategy in this study may be applicable to other oleaginous microalgae to boost biofuel production.

## Conclusion

In this study, *FAX1*/*FAX2/ABCA2*-OE were generated, which showed better traits for lipid accumulation than the parental line and previously reported individual FA transporter-OE. The co-expression of *FAX1*, *FAX2* and *ABCA2* enhanced the accumulation of TAG, total FA and membrane lipids under standard growth conditions. Moreover, we found that the increased expression of *FAX1*/*FAX2*/*ABCA2* enhanced the lipid biosynthesis, and accelerated the PUFA remobilization from membrane lipids to TAG by fine-tuning the key genes in lipid metabolism. This study provided valuable insights into facilitating the production of oils in microalgae, which may promote the production of microalgal biofuels with cost efficiency in the future.

## Methods

### Strains and culture conditions

The *Chlamydomonas* UVM4 strain [39] provided by Prof. Ralph Bock (Max-Planck Institute for Molecular Plant Physiology, Germany) was used as the parental strain for over-expressor generation. *ABCA2-*OE was generated in our previous study [16]. Unless otherwise stated, cells were routinely cultivated mixotrophically at 25 °C in Tris–acetate phosphate (TAP) medium [40], with 120 rpm shaking and constant illumination at 100 µmol m^−2^ s^−1^ [41]. For nitrogen deprivation, exponentially grown cells in TAP medium were centrifuged at 500 g for 5 min, and cell pellets were washed twice with nitrogen-depleted media (TAP-N) before finally being re-suspended in TAP-N for starvation experiments. Cells concentration and growth were monitored with automated Algae Counter (Countstar BioMarine).

### Construction of plasmids and transformation conditions

Genomic DNA of *Chlamydomonas* was isolated by the cetyltrimethyl ammonium bromide (CTAB) method as previously described [42]. The genomic DNA fragments coding for *FAX1* (Cre10.g421750) and *FAX2* (Cre08.g366000) were amplified using primers (FAX1-F and FAX1-R) and (FAX2-F and FAX2-R), respectively. The PCR reaction was carried out using the high fidelity KOD FX DNA Polymerase (Toyobo), according to the manufacturer’s instructions. The amplified DNA fragments of *FAX1* and *FAX2* were cloned as an *EcoR*I-*Xba*I fragment into the pChlamy_4 vector (Thermo Fisher) which contains the *ble* gene conferring zeocin resistance [41], generating pChlamy4-FAX1/FAX2 plasmid. The cDNA fragment of *ABCA2* was amplified using pChlamy4-cABCA2 plasmid [16] as template with primers (ABCA2-F and ABCA2-R), and was digested and subcloned into the pOpt-Clover-Paro plasmid [43] as an *EcoR*I-*Nde*I fragment to generate the *ABCA2* over-expression construct (pOpt-ABCA2-Paro). The sequences of all the primers used in this study are shown in Additional file 2: Table S1.

Before transformation, vectors were linearized with a single restriction enzyme *SspI* and purified using the MiniBEST DNA Fragment Purification Kit (Takara), according to the manufacturer’s protocols. Approximately 1.0 μg linearized plasmid DNA was used during each transformation experiment. The nuclear transformation was performed by electroporation as we previously described [41]. Briefly, *Chlamydomonas* cells were grown to approximately 1.5 × 10^6^ cells/mL in TAP medium. The exponentially grown cells (2.5 × 10^7^ cells) were harvested by centrifugation and suspended in 250 μL of TAP medium supplemented with 40 mM sucrose. The cell suspension was placed into a prechilled disposable electroporation cuvette with a 4-mm gap (Bio-Rad) for 5 min at 16 °C. Electroporation was performed by BioRad Gene Pulser Xcell with the following settings (voltage 500 V, Capacity 50 µF and Resistance 800 Ω). Electroporated cells were recovered with 5.0 mL of 40 mM sucrose in TAP medium for 16 h under the dim light (10 µmol m^−2^ s^−1^), and then plated on TAP agar plates containing single antibiotic (15 µg/mL zeocin) or dual antibiotics (15 µg/mL zeocin and 20 µg/mL paromomycin), and the plates were incubated under continuous light (50 µmol m^−2^ s^−1^) at 25 °C. Antibiotic-resistant colonies were visible after around 7 days.

### PCR-based determination of gene-positive transformants

The stable integration of *FAX1*, *FAX2* or *ABCA2* in the transformants was determined by PCR analysis of cell lysates, as we previously described [44, 45]. In brief, exponentially grown cells (5.0 × 10^6^) were resuspended in Tris–EDTA solution and incubated at 98 °C for 10 min. Aliquots (1.0 µL) of the supernatants from denatured cell lysates were then used as template for 20 µL PCR. For *FAX1* and *FAX2* gene positive screens, the primers (FAX1-F and FAX2-R) were used. For *ABCA2* gene positive PCR screens, the primers ABCA2-Rev5/Rbcs2 Pro and ABCA2-Forw5/Strp-tag-R were used to N- and C-terminal of expression cassette, respectively. The sequences of all the primers used in this study are shown in Additional file 2: Table S1.

### Nucleic acid extraction and expression level analysis

Total RNA was isolated as previously described [16, 41] using TRIzol reagent (Thermo Fisher), purified total RNA was treated with DNase I (Thermo Fisher) to remove the residual genomic DNA. For the first-strand cDNA was synthesized, 3.0 µg RNA was subjected to reverse transcription using PrimeScript 1st Strand cDNA Synthesis Kit (Takara), according to the manufacturer’s protocols. For RT-PCR, the PCR product of the housekeeping gene *RACK1* (Cre06.g278222) was employed as a loading control using previously reported primers [46, 47]. To estimate gene expression levels, real-time PCR (qRT-PCR) was performed on Applied Biosystems 7500 using TB Green Premix Ex Taq (Takara). Relative gene expression was analysed by the 2^−ΔΔ*C*T^ method [48], using *RACK1* as the reference gene. Primer sequences are listed in Additional file 1: Table S1.

### Lipid extraction and analyses

Total lipids were extracted by modified Bligh and Dyer method with modifications as we previously described [31, 41]. Briefly, the cells cultivated under N-replete condition at logarithmic phase (60 million of cells) or N-depleted condition for 2 days (20 million of cells) were harvested, and 1.0 mL of 1.0 mM EDTA in 0.15 M acetic acid was added to resuspend the pellets. The mixture was vortexed for 10 min, after addition of 3.0 mL methanol:chloroform (2:1, v/v) mixture. Then, 1.0 mL of chloroform and 0.8 mL of 0.88% (w/v) KCl were added and vortexed, and then centrifuged at 3000 rpm for 5 min. The lower chloroform phase was recovered. Cells were then extracted again with hexane, and the supernatant was combined with the previous chloroform extracts. Extracted lipids were dried under a stream of nitrogen and then re-dissolved in chloroform for non-polar lipid or polar lipid analysis by thin-layer chromatography (TLC). The non-polar lipids (mainly TAG) were separated on TLC plates (Merck) by a solvent mixture of hexane-diethyl ether-acetic acid (85:15:1, v/v/v), and the polar lipids were separated on TLC plates using chloroform–methanol-acetic acid–water (25:4:0.7:0.3, v/v/v/v) as development solvent [11]. The lipids separated on the plates were visualized by spraying with 0.05% (m/v) primulin (Sigma-Aldrich) in acetone/water (80/20, v/v). The TAG or polar lipid containing silica was recovered, and transmethylated into fatty acid methyl esters (FAMEs). For total fatty acids analysis, the protocol of direct transmethylation of each lipid was used as previously described [49, 50]. Quantification of FAMEs was performed by gas chromatography with flame ionization detector (GC-FID) fitted with an Agilent DB-23 (60 m × 0.25 mm × 0.25 μm) column, the parameters of detections were previously described [51]. Analysis was carried out using Agilent Chemstation software, and glycerol triheptadecanoate (TAG 51:0, 17:0/17:0/17:0, Sigma-Aldrich) was used as an internal standard to determine fatty acid recovery for quantification. For each treatment, three biological replicates (independent cultures) with three technical replicates were performed.

### Biomass determination

Biomass was determined by dry weight measurements as previously described [46, 52]. Briefly, the same concentration of cells (2.0 × 10^6^ cells/mL) were inoculated and cultivated. Subsequently, 10.0 mL of culture medium containing cells at logarithmic phase was collected on the preweighed Whatman GF/C filters (47 mm diameter) and dried overnight at 80 °C in an incubator. The dried biomass was then gravimetrically measured.

### Photosynthetic activity measurement

The maximal quantum conversion efficiency of PS II (*F*_v_*/F*_m_) of algal cells was measured by pulse amplitude-modulated (PAM) fluorometry (Water-PAM WALZ, Germany) as previously described [52, 53]. Briefly, the cells were dark-adapted for 10 min inside the spectrophotometer before the measurement of the maximal PS II quantum yield. Maximal PS II quantum yield was termed as *F*_v_*/F*_m_, where *F*_v_ represented the variation of chlorophyll fluorescence between maximal fluorescence (*F*_m_) induced by saturating pulse and initial fluorescence (*F*_0_) (*F*_m_*−F*_*0*_*/F*_m_). *F*_0_ was recorded under a weak light (27 μmol m^−2^ s^−1^, peaking at 650 nm) and *F*_m_ was under a saturating pulse (0.8 s) of red light (4000 μmol m^−2^ s^−1^, peaking at 660 nm).

### Statistical analysis

All the experiments in this study were performed at least three times. Cell samples were collected from three independent cultures (biological replicates) with three aliquots from the same culture (technical replicates) to obtain statistically data. The statistical significance of the differences was evaluated by Tukey’s honestly significant difference (HSD) test and Student’s *t*-test using SPSS 18.0.

### Accession numbers

Sequence data of *Chlamydomonas reinhardtii* genes presented in this article can be found in Phytozome (https://phytozome-next.jgi.doe.gov/) with gene identifications as: *FAX1* (Cre10.g421750), *FAX2* (Cre08.g366000), *ABCA2* (Cre14.g613950), *PDAT1* (Cre02.g106400), *PGD1* (Cre03.g193500), *DGAT1* (Cre01.g045903), *DGTT1* (Cre12.g557750), *DGTT2* (Cre09.g386912), *DGTT3* (Cre06.g299050), *DGTT4* (Cre03.g205050), *DGTT5* (Cre02.g079050) and *RACK1* (Cre06.g278222).

## Supplementary Information

**Additional file 1:** **Fig. S1.** PCR analysis of whole cell lysates from the FAX1/FAX2-OE and FAX1/FAX2/ABCA2-OE reveals that the transformants were stably transformed. **(a)** Colony PCR of FAX1/FAX2-OE using the primers (FAX1-F and FAX2-R) to amplify the full-length of DNA sequences of FAX1 and FAX2, and the predicted size of 2092 bp was detected. **(b, c)** Colony PCR of FAX1/FAX2/ABCA2-OE using the primers (ABCA2-Rev5/Rbcs2 Pro) and (ABCA2-Forw5/Strp-tag-R) to amplify the N- and C-terminal of the expression cassette, respectively. The predicted sizes of 484 bp and 381 bp were detected, respectively. M, DNA ladder. **Fig. S2.** Under nitrogen depleted condition (2 days), TAG content **(a)**, total fatty acids content **(b)**, and profiles of individual fatty acid content of total fatty acids (TFA) **(c)** in the transformants were analysed. The distinct letters labelled indicate the statistically significant difference by Tukey’s HSD test. **Fig. S3.** Total fatty acids content in the independent transformants of FAX1/FAX2/ABCA2 at exponential phase (day 2) under standard growth condition. The distinct letters labelled indicate the statistically significant difference by Tukey’s HSD test. **Fig. S4.** Alterations of membrane glycerolipid levels in the independent transformants of FAX1/FAX2/ABCA2 at exponential phase (day 2) under standard growth condition. **(a)** Total membrane lipid content. **(b)** Profile of individual membrane component contents. The total membrane lipid content referred to the total amount of major galactolipids (MGDG, DGDG and SQDG) and phospholipids (PE, PG and PI), and betaine lipid DGTS. MGDG, monogalactosyldiacylglycerol; DGDG, digalactosyldiacylglycerol; DGTS, diacylglycerol-N,N,N-trimethylhomoserine; SQDG, sulfoquinovosyldiacylglycerol; PG, phosphatidylglycerol; PE, phosphatidylethanolamine; PI, phosphatidylinositol. The distinct letters labelled indicate the statistically significant difference by Tukey’s HSD test. **Fig. S5.** Under standard growth condition at logarithmic phase, the profiles of individual fatty acids content of major thylakoid membrane MGDG **(a)**, DGDG **(b)**, and extraplastidic lipid DGTS **(c)** in the transformants were analysed. MGDG, monogalactosyldiacylglycerol; DGDG, digalactosyldiacylglycerol. The distinct letters labelled indicate the statistically significant difference by Tukey’s HSD test. **Fig. S6.** Analysis of the major contribution source for accumulation of TFA, TAG and membrane lipids in FAX1/FAX2/ABCA2-OE in relative to UVM4 under standard growth condition at logarithmic phase. **(a)** Contribution ratios of TAG and polar lipids (PL) to the accumulation of total fatty acids (TFA) in *FAX1/FAX2/ABCA2-OE* relative to UVM4, respectively. The contribution (%) is calculated as Con(%) = (cTAG_OE_ –cTAG_UVM4_)/(cTFA_OE_-cTFA_UVM4_) ×100. cTAG means the content of TAG, and cTFA means the content of total fatty acids **(b)** Contribution ratios of MGDG, DGDG and DGTS to PL accumulation in FAX1/FAX2/ABCA2-OE relative to UVM4, respectively. The contribution (%) is calculated as Con(%) = (iPL_OE_ – iPL_UVM4_)/(tPL_OE_-tPL_UVM4_) ×100. iPL means the content of individual polar lipids (MGDG, DGDG and DGTS), respectively, and tPL means the content of total polar lipids **(c)** Polyunsaturated FA levels in TFA, TAG, MGDG, DGDG, and DGTS in FAX1/FAX2/ABCA2-OE and UVM4, respectively. The polyunsaturated FA level (%) is calculated as (%) = cPUFA/cTFA×100. cPUFA means the content of total polyunsaturated FA, and cTFA total FA content. **(d)** Contribution ratios of the major PUFAs to TFA in FAX1/FAX2/ABCA2-OE relative to UVM4. The contribution (%) is calculated as Con(%) = (iPUFA_OE_ –iPUFA_UVM4_)/(cTFA_OE_-cTFA_UVM4_) ×100. iPUFA mean the content of 16:4 (4,7,10,13), 18:2 (9,12), 18:3 (9,12,15), and 18:3 (5,9,12), respectively. cTFA means total FA content. **(e)** Contribution ratios of the major PUFA for MGDG, DGDG and DGTS accumulation in FAX1/FAX2/ABCA2-OE relative to UVM4, respectively. The contribution (%) is calculated as Con(%) = (iPUFA_OE_–iPUFA_UVM4_)/(iPL_OE_-iPL_UVM4_) ×100. iPUFA are annotated as above. iPL mean the content of MGDG, DGDG and DGTS, respectively. **(f)** Contribution ratios of the major PUFA to TAG in FAX1/FAX2/ABCA2-OE relative to UVM4. The contribution (%) is calculated as Con(%) = (mPUFA_OE_ –mPUFA_UVM4_)/(cTAG_OE_-cTAG_UVM4_) ×100. mPUFA mean the content of 16:4 (4,7,10,13), 18:3 (9,12,15), and 18:3 (5,9,12), respectively. cTAG mean TAG contents. Asterisks indicate statistically significant changes compared to the parental line UVM4 by paired-sample Student’s t test (*P ≤ 0.05; **P ≤ 0.01).

**Additional file 2:** **Table S1. **All primer sequences used in this study.

## Data Availability

All data supporting the conclusions of this article are included within the article and in Additional files.

## Abbreviations

ABC: ATP-binding cassette
*Chlamydomonas*: *Chlamydomonas reinhardtii*
DAG: Diacylglycerol
FA: Fatty acids
TAG: Triacylglycerols
FAX: Fatty acid exporters
PUFA: Polyunsaturated fatty acids
ER: Endoplasmic reticulum
DGAT: Diacylglycerol acyltransferases
MGDG: Monogalactosyldiacylglycerol
DGDG: Digalactosyldiacylglycerol
DGTT: Type II diacylglycerol acyltransferases
DGTS: Diacylglyceryltrimethylhomoserine
PDAT1: Phospholipid:diacylglycerol acyltransferase 1
PGD1: Plastid galactoglycerolipid degradation 1
PS II: Photosystem II
SQDG: Sulfoquinovosyldiacylglycerol
PG: Phosphatidylglycerol
PE: Phosphatidylethanolamine
PI: Phosphatidylinositol
RACK1: Receptor of activated protein kinase C 1
