# Supplementary material for: Enhanced accumulation of oil through co-expression of fatty acid and ABC transporters in Chlamydomonas under standard growth conditions
Source: Biotechnol Biofuels Bioprod. 2022 May 20;15:54. doi: 10.1186/s13068-022-02154-6 (PMC9123788; doi:10.1186/s13068-022-02154-6)
Supplement: Supplementary file 1 — Additional file 1: Fig. S1. PCR analysis of whole cell lysates from the FAX1/FAX2-OE and FAX1/FAX2/ABCA2-OE reveals that the transformants were stably transformed. (a) Colony PCR of FAX1/FAX2-OE using the primers (FAX1-F and FAX2-R) to amplify the full-length of DNA sequences of FAX1 and FAX2, and the predicted size of 2092 bp was detected. (b, c) Colony PCR of FAX1/FAX2/ABCA2-OE using the primers (ABCA2-Rev5/Rbcs2 Pro) and (ABCA2-Forw5/Strp-tag-R) to amplify the N- and C-terminal of the expression cassette, respectively. The predicted sizes of 484 bp and 381 bp were detected, respectively. M, DNA ladder. Fig. S2. Under nitrogen depleted condition (2 days), TAG content (a), total fatty acids content (b), and profiles of individual fatty acid content of total fatty acids (TFA) (c) in the transformants were analysed. The distinct letters labelled indicate the statistically significant difference by Tukey’s HSD test. Fig. S3. Total fatty acids content in the independent transformants of FAX1/FAX2/ABCA2 at exponential phase (day 2) under standard growth condition. The distinct letters labelled indicate the statistically significant difference by Tukey’s HSD test. Fig. S4. Alterations of membrane glycerolipid levels in the independent transformants of FAX1/FAX2/ABCA2 at exponential phase (day 2) under standard growth condition. (a) Total membrane lipid content. (b) Profile of individual membrane component contents. The total membrane lipid content referred to the total amount of major galactolipids (MGDG, DGDG and SQDG) and phospholipids (PE, PG and PI), and betaine lipid DGTS. MGDG, monogalactosyldiacylglycerol; DGDG, digalactosyldiacylglycerol; DGTS, diacylglycerol-N,N,N-trimethylhomoserine; SQDG, sulfoquinovosyldiacylglycerol; PG, phosphatidylglycerol; PE, phosphatidylethanolamine; PI, phosphatidylinositol. The distinct letters labelled indicate the statistically significant difference by Tukey’s HSD test. Fig. S5. Under standard growth condition at logarithmic phase, [file 13068_2022_2154_MOESM1_ESM.docx]

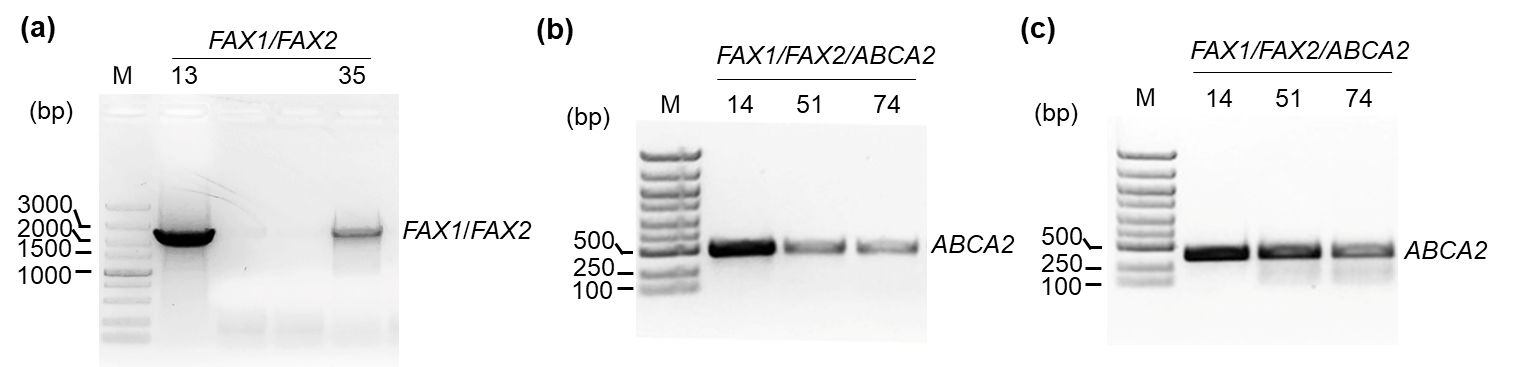


**Fig. S1.** PCR analysis of whole cell lysates from the *FAX1/FAX2-OE* and *FAX1/FAX2/ABCA2*-OE reveals that the transformants were stably transformed. **(a)** Colony PCR of *FAX1/FAX2-OE* using the primers (FAX1-F and FAX2-R) to amplify the full-length of DNA sequences of *FAX1* and *FAX2,* and the predicted size of 2092 bp was detected. **(b, c)** Colony PCR of *FAX1/FAX2/ABCA2-OE* using the primers (ABCA2-Rev5/Rbcs2 Pro) and (ABCA2-Forw5/Strp-tag-R) to amplify the N- and C-terminal of the expression cassette, respectively. The predicted sizes of 484 bp and 381 bp were detected, respectively. M, DNA ladder.


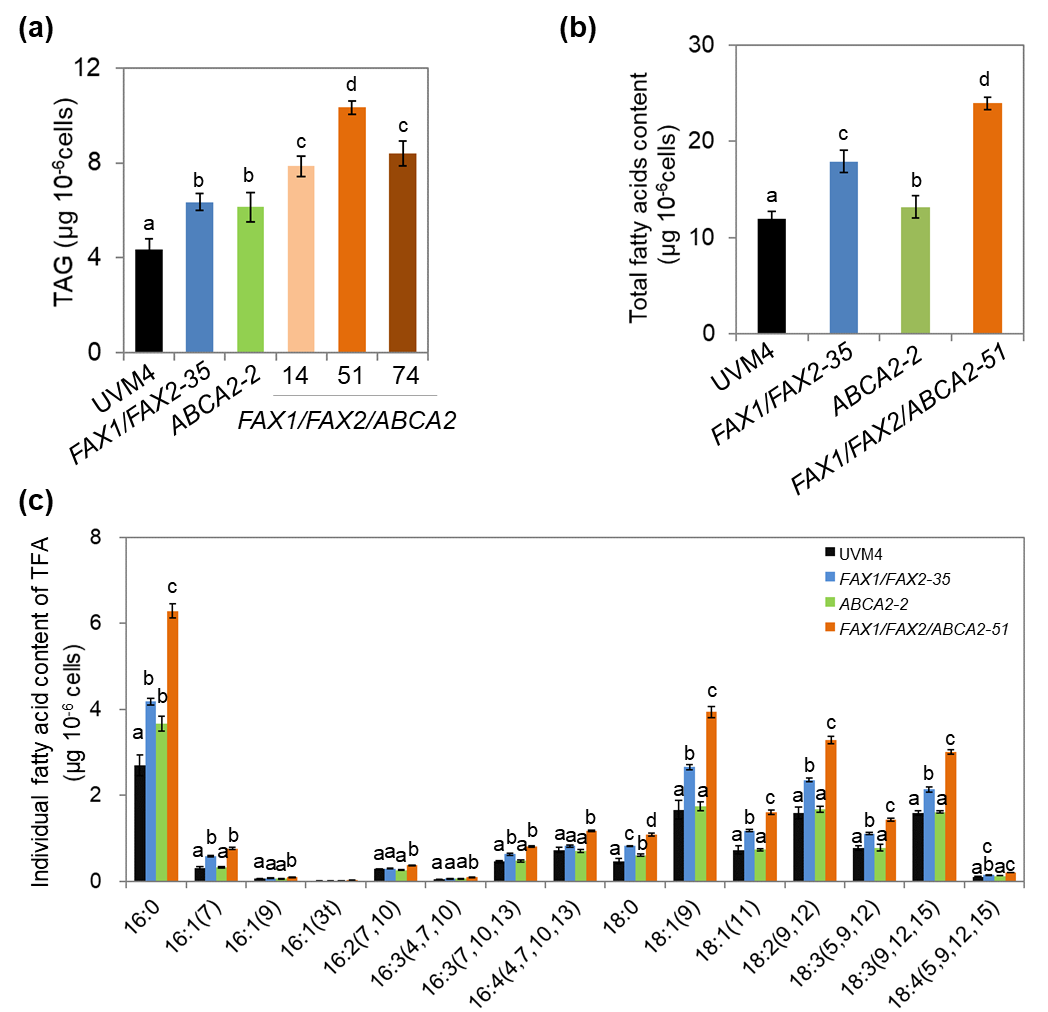


**Fig. S2.** Under nitrogen depleted condition (2 days), TAG content **(a)**, total fatty acids content **(b)**, and profiles of individual fatty acid content of total fatty acids (TFA) **(c)** in the transformants were analyzed. The distinct letters labelled indicate the statistically significant difference by Tukey’s HSD test.

**
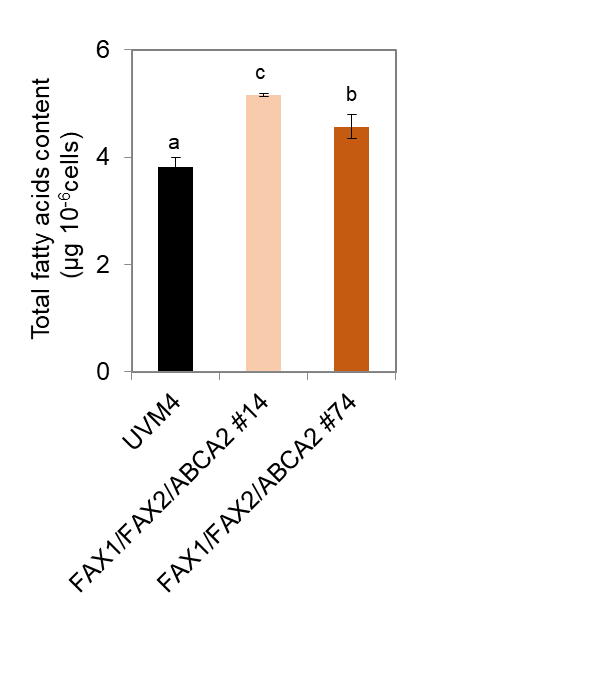
**

**Fig. S3. Total fatty acids content in the independent transformants of *FAX1*/*FAX2/ABCA2* at exponential phase (day 2) under standard growth condition.** The distinct letters labelled indicate the statistically significant difference by Tukey’s HSD test.


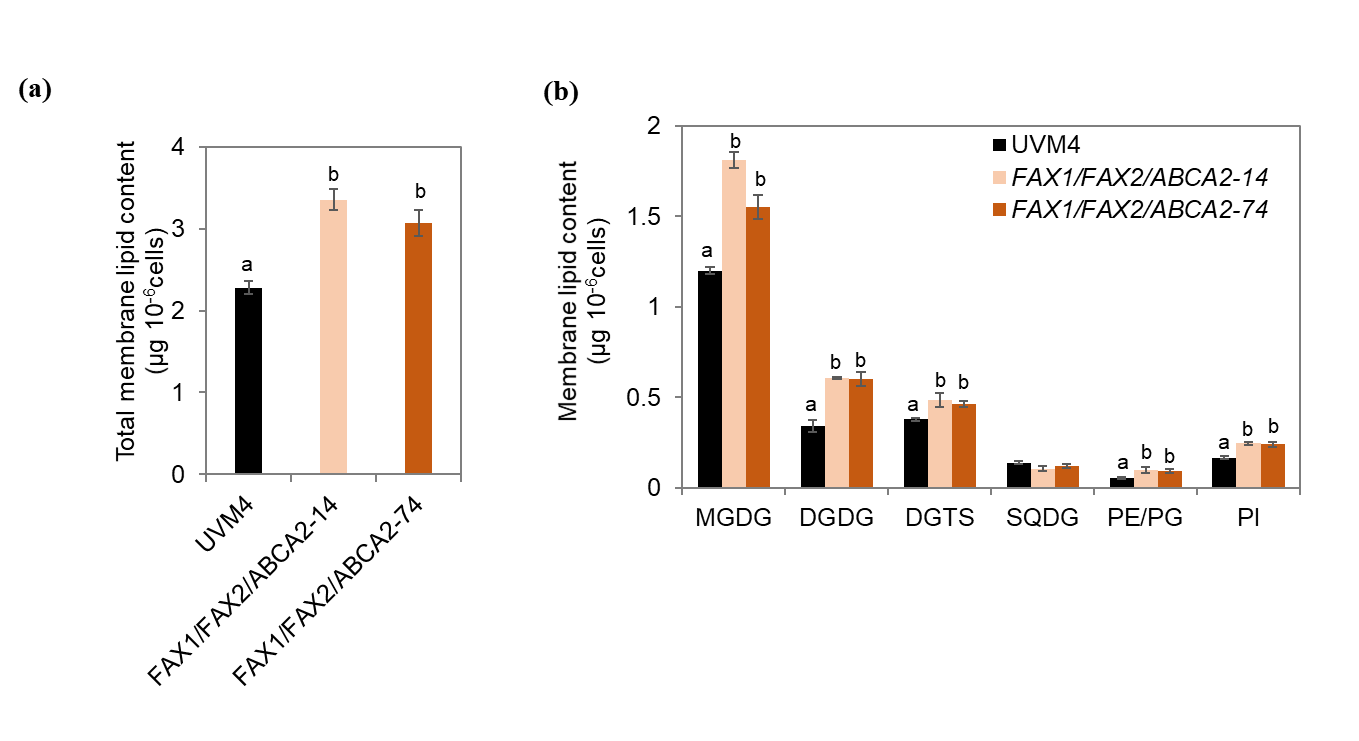


**Fig. S4. Alterations of membrane glycerollipid levels in the independent transformants of *FAX1*/*FAX2/ABCA2* at exponential phase (day 2) under standard growth condition.** **(a)** Total membrane lipid content. **(b)** Profile of individual membrane component contents. The total membrane lipid content referred to the total amount of major galactolipids (MGDG, DGDG and SQDG) and phospholipids (PE, PG and PI), and betaine lipid DGTS. MGDG, monogalactosyldiacylglycerol; DGDG, digalactosyldiacylglycerol; DGTS, diacylglycerol-*N*,*N*,*N*-trimethylhomoserine; SQDG, sulfoquinovosyldiacylglycerol; PG, phosphatidylglycerol; PE, phosphatidylethanolamine; PI, phosphatidylinositol. The distinct letters labelled indicate the statistically significant difference by Tukey’s HSD test.


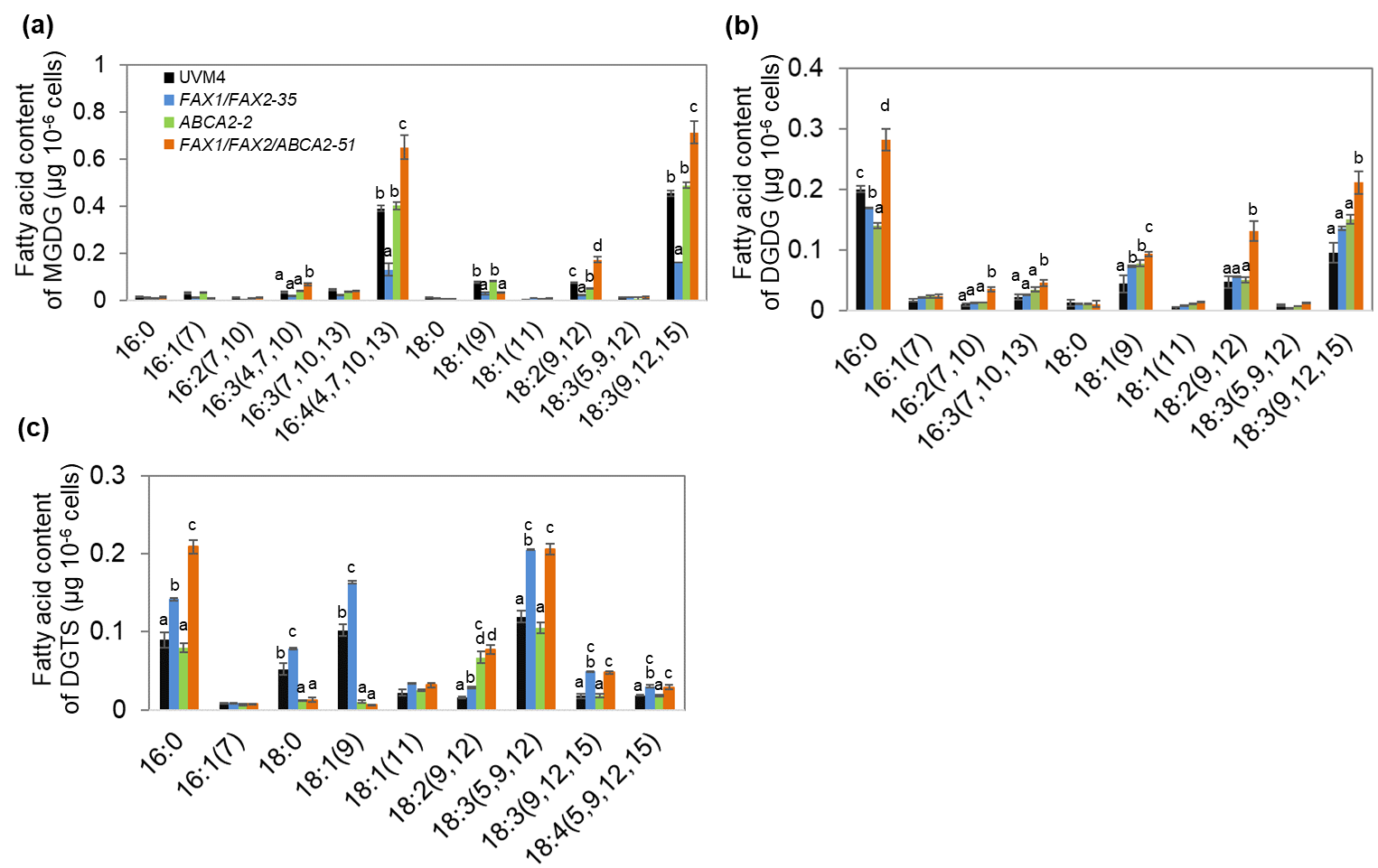


**Fig. S5.** Under standard growth condition at logarithmic phase, the profiles of individual fatty acids content of major thylakoid membrane MGDG **(a)**, DGDG **(b)**, and extraplastidic lipid DGTS **(c)** in the transformants were analyzed. MGDG, monogalactosyldiacylglycerol; DGDG, digalactosyldiacylglycerol. The distinct letters labelled indicate the statistically significant difference by Tukey’s HSD test.


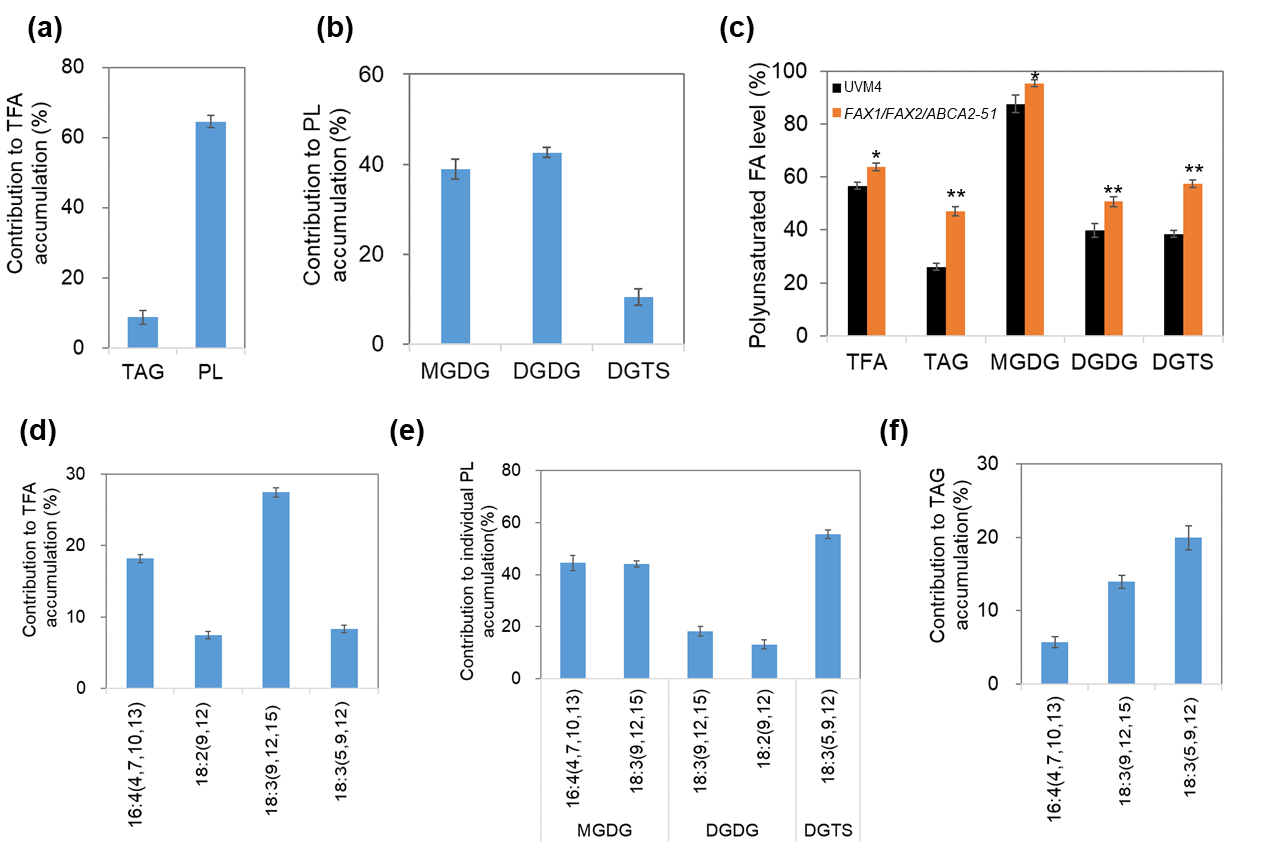


**Fig. S6**. Analysis of the major contribution source for accumulation of TFA, TAG and membrane lipids in *FAX1/FAX2/ABCA2*-OE in relative to UVM4 under standard growth condition at logarithmic phase. **(a)** Contribution ratios of TAG and polar lipids (PL) to the accumulation of total fatty acids (TFA) in *FAX1/FAX2/ABCA2-OE* relative to UVM4, respectively. The contribution (%) is calculated as Con(%) = (cTAG_OE_ –cTAG_UVM4_)/(cTFA_OE_－cTFA_UVM4_) ×100. cTAG means the content of TAG, and cTFA means the content of total fatty acids **(b)** Contribution ratios of MGDG, DGDG and DGTS to PL accumulation in *FAX1/FAX2/ABCA2-OE* relative to UVM4, respectively. The contribution (%) is calculated as Con(%) = (iPL_OE_ – iPL_UVM4_)/(tPL_OE_－tPL_UVM4_) ×100. iPL means the content of individual polar lipids (MGDG, DGDG and DGTS), respectively, and tPL means the content of total polar lipids **(c)** Polyunsaturated FA levels in TFA, TAG, MGDG, DGDG, and DGTS in *FAX1/FAX2/ABCA2-OE* and UVM4, respectively. The polyunsaturated FA level (%) is calculated as (%) = cPUFA/cTFA×100. cPUFA means the content of total polyunsaturated FA, and cTFA total FA content. **(d)** Contribution ratios of the major PUFAs to TFA in *FAX1/FAX2/ABCA2-OE* relative to UVM4. The contribution (%) is calculated as Con(%) = (iPUFA_OE_ –iPUFA_UVM4_)/(cTFA_OE_－cTFA_UVM4_) ×100. iPUFA mean the content of 16:4(4,7,10,13), 18:2(9,12), 18:3(9,12,15), and 18:3(5,9,12), respectively. cTFA means total FA content. **(e)** Contribution ratios of the major PUFA for MGDG, DGDG and DGTS accumulation in *FAX1/FAX2/ABCA2-OE* relative to UVM4, respectively. The contribution (%) is calculated as Con(%) = (iPUFA_OE_ –iPUFA_UVM4_)/(iPL_OE_－iPL_UVM4_) ×100. iPUFA are annotated as above. iPL mean the content of MGDG, DGDG and DGTS, respectively. **(f)** Contribution ratios of the major PUFA to TAG in *FAX1/FAX2/ABCA2-OE* relative to UVM4. The contribution (%) is calculated as Con(%) = (mPUFA_OE_ –mPUFA_UVM4_)/(cTAG_OE_－cTAG_UVM4_) ×100. mPUFA mean the content of 16:4(4,7,10,13), 18:3(9,12,15), and 18:3(5,9,12), respectively. cTAG mean TAG contents. Asterisks indicate statistically significant changes compared to the parental line UVM4 by paired-sample Student’s *t* test (^*^*P* ≤ 0.05; ^**^*P* ≤ 0.01).
