# Supplementary material for: Enhanced accumulation of oil through co-expression of fatty acid and ABC transporters in Chlamydomonas under standard growth conditions
Source: Biotechnol Biofuels Bioprod. 2022 May 20;15:54. doi: 10.1186/s13068-022-02154-6 (PMC9123788; doi:10.1186/s13068-022-02154-6)
Supplement: Supplementary file 2 — Additional file 2: Table S1. All primer sequences used in this study. [file 13068_2022_2154_MOESM2_ESM.docx]

**Table S1. All primer sequences used in this study.**

| **Usage** | **Primer name** | **Sequence (5’-3’)** |
| --- | --- | --- |
| PCR for gene amplification | FAX1-F | CCGGAATTCGCTGCTTCCCTGCTGCAACG |
|  | FAX1-R | CGCGGATCCCTCGGCCTTGCCGGCGGC |
|  | FAX2-F | CGCGGATCCTATGACTTTTGCTTTTCGCCCATTT |
|  | FAX2-R | GCTCTAGACCGTGCGCCTTGCCGTGC |
|  | ABCA2-F | AACCGCCATATGATGGCTCGACTTTCTTG |
|  | ABCA2-R | AACTAGGAATTCTCAGTTGCGGGAGTCCA |
| Chlamydomonas colonies PCR | FAX1-F | CCGGAATTCGCTGCTTCCCTGCTGCAACG |
|  | FAX2-R | GCTCTAGACCGTGCGCCTTGCCGTGC |
|  | Rbcs2 pro | CCAATGCAAGCAGTTCGCATGC |
|  | ABCA2-Rev5 | CGGTGTTGTTGCTGTCATTCGCT |
|  | ABCA2-Forw5 | ATCATCATGGACTGCACGGAGCT |
|  | Strp-tag-R | TTACTTCTCGAACTGCGGGTGGCT |
| RT-PCR | FAX1-F | CCGGAATTCGCTGCTTCCCTGCTGCAACG |
|  | FAX2-R | GCTCTAGACCGTGCGCCTTGCCGTGC |
|  | CBLP-F1 | CTTCTCGCCCATGACCAC |
|  | CBLP-R1 | CCCACCAGGTTGTTCTTCAG |
|  | ABCA2-F1 | CATGTTCAGCTTCGTCATCCAG |
|  | ABCA2-R1 | TCTTGTAGTAGTCGGGCGTGTA |
| qRT-PCR | RACK1-F1 | CTTCTCGCCCATGACCAC |
|  | RACK1-R1 | CCCACCAGGTTGTTCTTCAG |
|  | PDAT1-F | CTGCCTCGCCTCAATGCCTACC |
|  | PDAT1-R | CAGCAGGCCGTTATAGGCGCTG |
|  | DGAT1-F | GCTCAACGTGCTAGCAGAGCTGA |
|  | DGAT1-R | CCGTATGGCTGGGAAGTAGACGT |
|  | PGD1-F | AGCCAGCTATTGTCGCACTT |
|  | PGD1-R | CAAGAAATCCGCTGACATCCGT |
|  | CrABCA2-F1 | CATGTTCAGCTTCGTCATCCAG |
|  | CrABCA2-R1 | TCTTGTAGTAGTCGGGCGTGTA |
|  | CrDGTT1-F | CGGGCTCTAAAGATGACGCCAACT |
|  | CrDGTT1-R | CGTGCGGGTGGCTCACGAATATAT |
|  | CrDGTT2-F | CGCACACCATGTAAATGTTGCGGC |
|  | CrDGTT2-R | TTTGGCAACTCTCAGGTGCTGGAC |
|  | CrDGTT3-F | CTGAAGAAGGGCAGTGTGGCGGT |
|  | CrDGTT3-R | CCAGCACTTGGCTCTGACCGAAGT |
|  | CrDGTT4-F | GTCAGCGCCATCTACACCTCGGT |
|  | CrDGTT4-R | ACACTGAACTGCACGAACCGCTC |
|  | CrDGTT5-F | GCTGAAAGGCCCGAAGGACGAT |
|  | CrDGTT5-R | AGGATCTTGCTGTTGCCCATGTGG |
